# Tryptophan to Tryptophan Hole Hopping in an Azurin Construct

**DOI:** 10.1021/acs.jpcb.3c06568

**Published:** 2023-12-25

**Authors:** Martin Melčák, Filip Šebesta, Jan Heyda, Harry B. Gray, Stanislav Záliš, Antonín Vlček

**Affiliations:** † J. Heyrovský Institute of Physical Chemistry, Czech Academy of Sciences, Dolejškova 3, CZ-182 23 Prague, Czech Republic; ‡ Department of Physical Chemistry, University of Chemistry and Technology Prague, Technická 5, CZ-166 28 Prague, Czech Republic; § Department of Chemical Physics and Optics, Faculty of Mathematics and Physics, Charles University, Ke Karlovu 3, CZ-121 16 Prague, Czech Republic; ∥ Beckman Institute, California Institute of Technology, Pasadena, California 91125, United States; ⊥ Department of Chemistry, Queen Mary University of London, London E1 4NS, U.K.

## Abstract

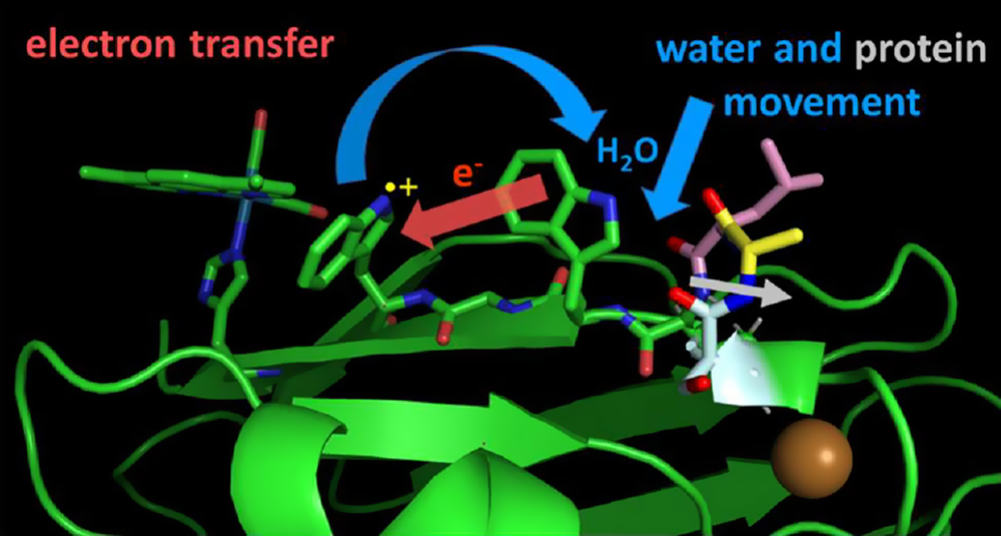

Electron transfer (ET) between neutral and cationic tryptophan residues in the azurin construct [Re^I^(H126)(CO)_3_(dmp)](W124)(W122)Cu^I^ (dmp = 4,7-Me_2_-1,10-phenanthroline) was investigated by Born–Oppenheimer quantum-mechanics/molecular mechanics/molecular dynamics (QM/MM/MD) simulations. We focused on W124^•+^ ← W122 ET, which is the middle step of the photochemical hole-hopping process *Re^II^(CO)_3_(dmp^•–^) ← W124 ← W122 ← Cu^I^, where sequential hopping amounts to nearly 10,000-fold acceleration over single-step tunneling (*ACS Cent. Sci*. **2019**, *5*, 192–200). In accordance with experiments, UKS-DFT QM/MM/MD simulations identified forward and reverse steps of W124^•+^ ↔ W122 ET equilibrium, as well as back ET Re^I^(CO)_3_(dmp^•–^) → W124^•+^ that restores *Re^II^(CO)_3_(dmp^•–^). Strong electronic coupling between the two indoles (≥40 meV in the crossing region) makes the productive W124^•+^ ← W122 ET adiabatic. Energies of the two redox states are driven to degeneracy by fluctuations of the electrostatic potential at the two indoles, mainly caused by water solvation, with contributions from the protein dynamics in the W122 vicinity. ET probability depends on the orientation of Re(CO)_3_(dmp) relative to W124 and its rotation diminishes the hopping yield. Comparison with hole hopping in natural systems reveals structural and dynamics factors that are important for designing efficient hole-hopping processes.

## Introduction

Electron (hole) hopping through chains of aromatic amino acid residues (tryptophan, tyrosine) accelerates charge transport in proteins.^1−4^ Hole hopping serves to deliver oxidizing equivalents to enzyme active sites in class Ia ribonucleotide reductases,^5^ regenerates the starting flavin oxidation state in photolyases and cryptochromes,^6−9^ and protects cytochrome P450 from self-destruction in the absence of substrates by channeling holes to the protein surface where they can be disarmed by cellular reductants.^1,10−12^ Hopping pathways are selective and directional, whereby the hole is delivered to its target despite the presence of off-path Trp-Tyr residues lying within relatively short hole-transfer distances. Clearly, efficient hopping pathways are defined not only by distances between hopping sites but also by redox-potential gradients that have been evolution-optimized in naturally occurring enzymes.^1^

**Scheme 1:**
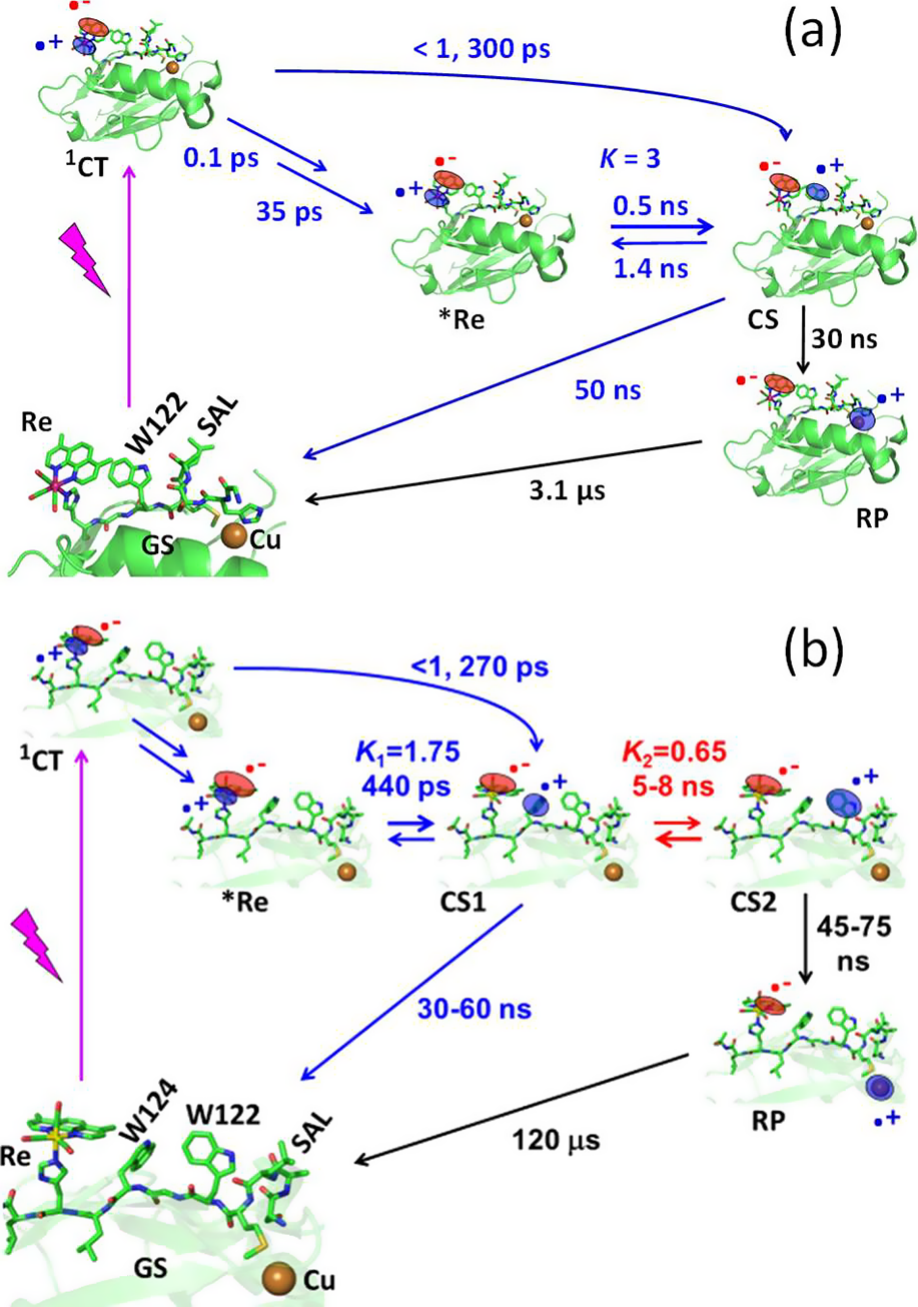
Summary of Experimental Data Top (a): **Re124W122Cu**^**I**^. Rate and equilibrium constants were obtained by fitting to lumininescence, visible transient absorption and TRIR kinetics.^13^ Bottom (b): **Re126W124W122Cu**^**I**^. Rate and equilibrium constants were obtained by modeling to match lumininescence decay and transient absorption kinetics.^15^ The <1 and 270 ps ET reactions occur concomitantly with ISC and ^1^CT relaxation. *Re decay to the ground state (1.15 μs) was omitted for clarity. CS1 = Re^I^(H126)(CO)_3_(dmp^•–^)(W124^•+^)(W122)Cu^I^. CS2 = Re^I^(H126)(CO)_3_(dmp^•–^)(W124)(W122^•+^)Cu^I^. Reaction product RP = Re^I^(H126)(CO)_3_(dmp^•–^)(W124)(W122)Cu^II^. SAL stands for the S118A119L120 segment. The reduced sensitizer Re^I^(H126)(CO)_3_(dmp^•–^) in CS1, CS2, and RP is abbreviated **Re**^**–**^ in the text.

Introducing artificial hopping pathways and chromophores into protein mutants and investigating charge transport mechanisms could reveal design principles that would lead to systems capable of efficient photochemical charge separation for light energy conversion and/or photocatalysis. The first such system was a *Pseudomonas aeruginosa* azurin mutant abbreviated **Re124W122Cu**^**I**^ (Scheme 1a). In this mutant, all naturally occurring Trp and Tyr residues were replaced by phenylalanine, and a Q124H mutation was introduced to provide a covalent histidine binding site for the Re^I^(H124)(CO)_3_(dmp)^+^ photooxidant (abbreviated **Re**; dmp = 4,7-Me_2_-1,10-phenanthroline), and a K122W mutation placed a Trp residue between the photooxidant and Cu^I^.^13^ Photoexcitation of the **Re** chromophore (*Re, a mixed ^3^MLCT/ππ*-dmp excited state^14^) was followed by fast W122 oxidation that exhibited multiphase kinetics (<1 ps, ∼300 ps, ∼500 ps). Cu^I^ was then oxidized in a second step by ∼30 ns hole transfer from W122^•+^ (Scheme 1a). With Re and Cu atoms separated by 19.4 Å, hopping through W122 accelerated Cu^I^ oxidation over 100-fold relative to *Re ← Cu^I^ single-step electron tunneling. Importantly, Cu^I^ photooxidation was not observed for mutants containing Lys, Tyr, or Phe at the W122 position.^13^

Extending the hopping system by shifting **Re** two sites farther away to H126, and introducing two Trp residues into the **Re**···Cu^I^ pathway by T124W and K122W mutations afforded **Re126W124W122Cu**^**I**^ (Scheme 1b). In this double-Trp mutant, Cu^I^ was photoxidized in ca. 80 ns by three-step *Re ← W124 ← W122 ← Cu^I^ ET over a distance of 22.9 Å.^15^ We found that optical excitation of **Re** in **Re126W124W122Cu**^**I**^ triggered (ultra)fast multiphase W124 oxidation, affording the CS1 state Re^I^(H126)(CO)_3_(dmp^•–^)(W124^•+^)(W122)Cu^I^. The second hop (W124^•+^ ← W122) produced the CS2 state Re^I^(H126)(CO)_3_(dmp^•–^)(W124)(W122^•+^)Cu^I^ in 5–8 ns. The final step, W122^•+^ ← Cu^I^ ET, occurred in 45–75 ns. Of interest is that hopping through two tryptophans in **Re126W124W122Cu**^**I**^ is roughly 10,000-fold faster than *Re ← Cu^I^ single-step tunneling. This huge kinetics advantage comes at a price, namely, a relatively low charge-separation yield that can be traced to the slightly energetically uphill (+11 meV) second ET step (W124^•+^ ← W122, the CS1 → CS2 conversion).^15^ This behavior is very different from that of photolyases, where the hole propagates downhill through a chain of three closely spaced tryptophans.^6−8^ Understanding differences between hole hopping through artificially constructed tryptophan pathways in azurins and evolution-optimized pathways in photolyases could reveal factors critical for efficient long-range charge separation and guide the design of de novo ET systems and photoenzymes.

Kinetics and spectroscopic studies, together with molecular dynamics simulations, could shed new light on the polypeptide and solvent molecular motions that drive individual ET steps. In addition, we could address the question of the extent to which the hole is (de)localized over the hopping pathway. Our previous theoretical investigation^14^ of tryptophan oxidation by *Re in **Re124W122Cu**^**I**^ (and **Re126W124W122Cu**^**I**^) employed TDDFT quantum mechanics/molecular mechanics/molecular dynamics (QM/MM/MD) to follow the time-evolution of a set of low-lying triplet excited states that included *Re as well as CS (CS1). Their energies were calculated relative to those of the singlet ground state (GS). We aimed to unravel structural, solvational, and dynamical factors that bring *Re and CS (CS1) states to degeneracy and lead to ET that was manifested by an abrupt change of charge distribution between *Re(CO)_3_(dmp)^+^ and the proximal indole. We have demonstrated that electronic coupling is relatively strong, fluctuating with the dmp/indole orientation and distance as well as with the charge distribution within *Re(CO)_3_(dmp)^+^. We concluded that this ET step is adiabatic, driven mainly by fluctuations of water molecules around the *Re···W unit.^14^ Increasing solvation of the proximal tryptophan was singled out as the main factor enabling ET and stabilizing the CS (CS1) state.^14^

**Figure 1:**
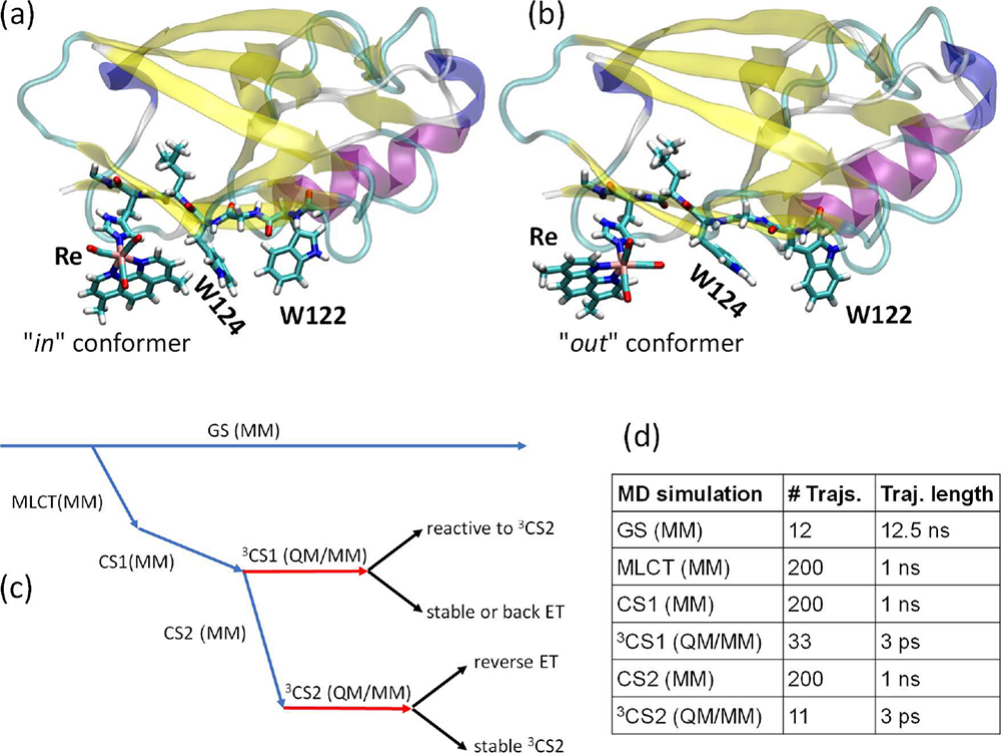
Top: (a) **Re126W124W122Cu**^**I**^ crystal structure (PDB: 6MJS) where **Re** assumes the ″*in*″ orientation. (b) Typical ″*out*″ structure taken at 8 ns from the MM/MD trajectory shown in Figure S1, second row, third column. (The occurrence of ″*in*″ and ″*out*″ conformers emerged from MM/MD, see the text.) The QM-described part is shown as sticks. The rest of the system, including all water molecules and 2 Na^+^ ions (to maintain electroneutrality), was treated classically. Bottom (c): Multiscale procedure that was employed to simulate CS1/CS2 conversion in **Re126W124W122Cu**^**I**^. Blue and red arrows depict classical (MM) and QM/MM MD trajectories, respectively. Black indicates subsequent analyses. (d) Numbers and lengths of each kind of trajectory. The computational procedure followed the experimentally determined sequence of reaction steps proceeding from the ground state to the *Re excited state (modeled as the MLCT) and then to CS1. 33 MM/MD-stabilized CS1 structures served as starting points for CS1 QM/MM/MD trajectories. Some of the CS1 MM/MD trajectories were followed by CS2 MM/MD simulations and then by QM/MM/MD to characterize the CS2 state.

In the present work, we proceeded to the second hopping step (W124^•+^ ← W122) that interconverts the ^3^CS1 and ^3^CS2 states of **Re126W124W122Cu**^**I**^. Excited-state ^3^CS2 energies calculated by TDDFT with the singlet reference ground state turned out to be unrealistically low, owing to vastly different charge distribution and solvation in either CS state compared to the GS reference. Hence, we have designed a QM/MM/MD simulation protocol (Figure 1) where the unrestricted Kohn–Sham (UKS) approach^16,17^ provided temporal evolution of the charge distribution in the lowest triplet state. Indeed, some of the simulations displayed a near-complete hole shift from W124^•+^ to W122. Their analysis then revealed fluctuations of the molecular structure, solvent, electron-density distribution, electronic coupling, and electrostatic potentials that drive the CS1 and CS2 states over the energy barrier, enabling the hopping process.

## Model and Simulation Methodology

### System Definition

For the purpose of simulations, the solvated **Re126W124W122Cu**^**I**^ system was divided into quantum (QM) and classical (MM) parts (Figure 1a,b). The QM part consisted of **Re**^**–**^, both tryptophan residues W124 and W122 (one of them bearing a single positive charge) and connecting L125 and G123 residues. It was terminated by linking-H atoms that were attached to C_α_ atoms of the L127 and M121 protein backbone. The classical part comprised the rest of the protein, 6683 water molecules, and two Na^+^ ions to make the system electroneutral.

### Computational Methodology

**Figure 2:**
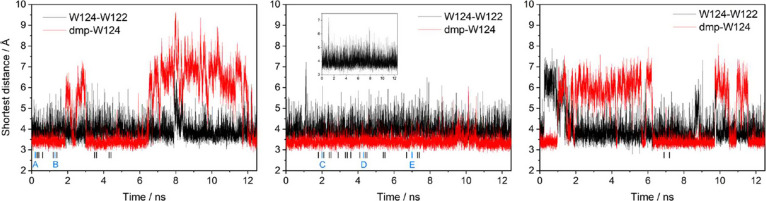
Selected ground-state MM/MD trajectories of **Re126W124W122Cu**^**I**^. Black: the shortest distance between heavy atoms of W122 and W124 indole rings. Red: the shortest distance between heavy atoms of W124 and dmp. Inset in the middle trajectory shows the extent of indole–indole distance fluctuations. This trajectory was used to calculate the mean values reported in the text. Bars at the bottom show starting points of MM/MD CS1 simulations that were followed by QM/MM/MD runs. They were selected randomly from ground-state ″*in*″ configurations. The full set of calculated GS trajectories is displayed in Figure S1.

The simulation procedure is shown in Figure 1c and is described in the legend. Further details are provided in the Supporting Information Sections S1.1.–S1.3.. In brief, simulations started by calculating 12 classical 12.5 ns-long MM/MD trajectories of the ground-state system. At 200 randomly selected points, the system was propagated to the lowest triplet metal to ligand charge transfer (^3^MLCT) state of the Re chromophore (*Re) by changing the force field, and 200 MLCT MM/MD simulations were run for 1 ns. Then, the force field was changed to describe the Re^I^(H126)(CO)_3_(dmp^•–^)(W124^•+^)(W122) CS1 state and simulations continued for another 1 ns. 33 selected final structures were taken as starting points for 3 ps–long QM/MM/MD simulations of the triplet CS1 state (see Figure 2 for the selection). In total, we have obtained 33 such QM/MM/MD CS1 trajectories (starting times indicated in Figure 2), which were sorted according to their outcomes (ET reactive/unreactive or undergoing reverse ET to *Re). We assumed that 3 ps is sufficient to identify reactive trajectories that start close to the *Re/CS1 crossing point. Their analysis then revealed structural and dynamic ET-facilitating factors. To characterize CS2 independently, the CS1 MM/MD trajectories were continued after 1 ns with parametrization pertinent to the CS2 charge distribution. Out of 200 CS2 MM/MD final structures, 11 were randomly selected for the CS2 QM/MM/MD simulations.

MD simulations of ^3^CS1 and ^3^CS2 excited states of **Re126W124W122Cu**^**I**^ in protein and solvent media were performed at the QM/MM level in the Terachem 1.9^18,19^ – Amber 14^20^ framework. The QM part of MD simulations described the lowest triplet state by the UKS formalism,^16,17^ using the PBE0 functional^21,22^ with the D3 dispersion correction.^23^ Test calculations with a long-range-corrected functional CAM-B3LYP^24^ led to unrealistically large energy separations but correctly reproduced electron-density distributions associated with CS1 and CS2 structures established with PBE0 (Supporting Information, Section S1.4.).

Electronic couplings between W122 and W124 indoles were calculated for charge-localized states CS1 and CS2 using configuration interactions with constrained DFT (CDFT-CI)^25^ and absolutely localized molecular orbitals (ALMO)^26^ at series of snapshots of QM/MM/MD trajectories employing Q-Chem 6.0 software^27^ with the PBE0-D3 functional. Test calculations with the long-range corrected CAM-B3LYP functional gave comparable |*H*_ab_| values (Supporting Information, Section S1.5. and Figure S27).

MM/MD simulations of the ground- and the lowest ^3^MLCT state utilized previously derived MM parameters.^14^ Sets of MM parameters for ^3^CS1 and ^3^CS2 in a solvated-protein environment were based on atomic charges that were calculated separately (QM) for Re^I^(H126)(CO)_3_(dmp^•–^)(W124^•+^)(W122) (^3^CS1) and Re^I^(H124)(CO)_3_(dmp^•–^)(W124)(W122^•+^) (^3^CS2) structurally optimized within the environment of solvated azurin (Supporting Information, Section S1.8.). Truhlar’s CM5 population analysis^28^ was used to determine atomic charges instead of the standard RESP procedure^29^ that led to an unrealistic (overpolarized) charge distribution at the Re chromophore. Snapshots from GS, MLCT, and CS1 and CS2 excited-state MM/MD trajectories provided initial positions and velocities for subsequent QM/MM/MD simulations. The CS1/CS2 crossing was monitored by charge and spin variations at the two indoles during the QM/MM/MD trajectories.

## Results

### Ground-State MM/MD Trajectories

The shortest non-hydrogen atom–atom distances between the two indoles rapidly fluctuated around the mean value of 3.40 Å with an amplitude up to about 2 Å. They occasionally shot up to 6–7 Å, but such structures were rather short-lived. The two indoles were approximately T-oriented. The relative orientation of Re(CO)_3_(dmp)^+^ and W124 switched frequently between two conformations, owing to Re(CO)_3_(dmp)^+^ rotation around the Re–N(H126) bond (Figures 2 and S1; typical structures are shown in Figures 1a,b and S3). The ″*in*″ conformation is similar to that found^13^ in the crystal. The dmp ligand was calculated close to W124, fluctuating nearly symmetrically around a 3.4 Å mean distance within a ca. 1 Å range. The ″*out*″ conformation spanned much larger dmp-W124 distances (6–7 Å) and the dmp ligand was oriented away from the indole. One of the two equatorial CO ligands pointed toward W124 at a distance of about 3 Å, in contrast with 6–7 Å in the ″*in*″ conformer (Figures S2 and S3). The other two COs pointed toward a neighboring β-sheet segment. The indole–indole distances were comparable to those in the ″*in*″ conformation. Overall, the system spent 63% of the total simulation time in the ″*in*″ form. Either conformation could last for several nanoseconds before turning to the other one.

### CS1 State

**Figure 3:**
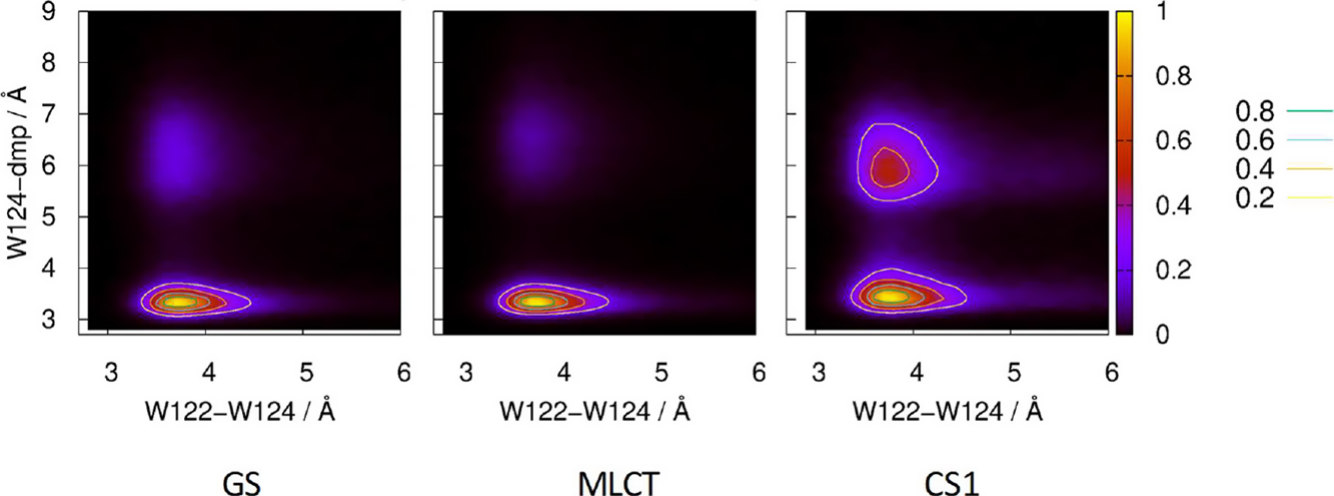
Probability distributions of W124-dmp and indole–indole distances in the ground, MLCT, and CS1 states calculated from 12 × 12.5 ns GS, 180 × 1 ns MLCT, and 180 × 1 ns CS1 MM/MD trajectories. Majority of GS and MLCT structures occurs in a relatively limited range of ″*in*″ conformations. The fraction of ″*out*″ structures is the largest in CS1. Figure S4 shows cases of ″*in*″ to ″*out*″ conformational changes and/or indole–indole elongations along 1 ns MM/MD CS1 trajectories.

To simulate CS1 structures and dynamics, we chose a set of GS structures (Figure 2), on which we ran MM/MD simulations first with MLCT and then with CS1 parametrizations. Starting structures for CS1 MM/MD were limited to the ″*in*″ conformation since CS1 formation by *Re ← W124 ET requires close contact between dmp and W124.^14^ Classical MLCT trajectories were rather stable, in contrast with CS1 where **Re**^**–**^ rotation to the ″*out*″ form occurred frequently within 1 ns classical simulations (Figures 3 and S4). On longer trajectories, the ″*out*″ conformation occasionally reverted for short times to ″*in*″ (Figure S4).

**Figure 4:**
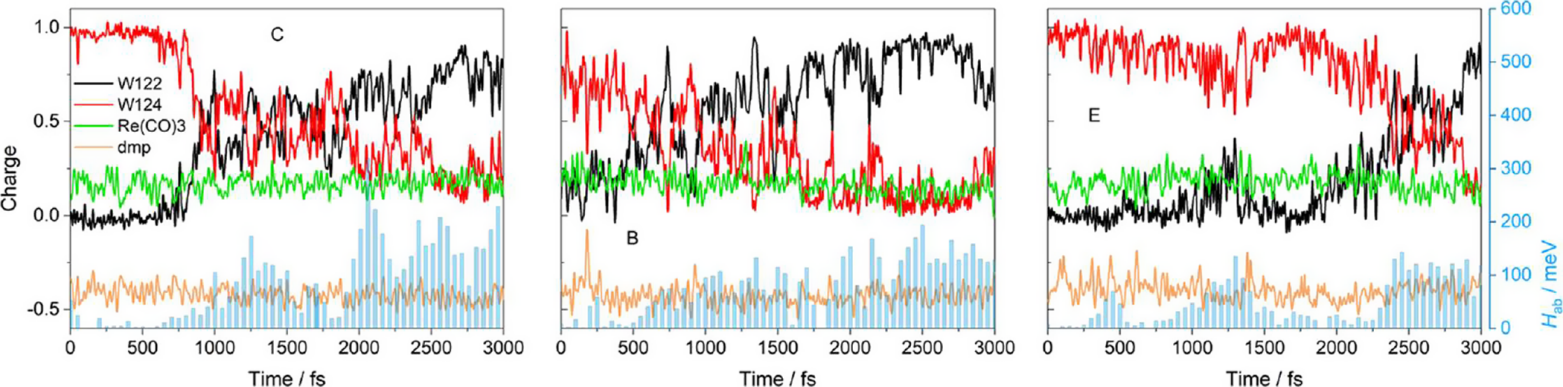
Evolution of Mulliken charges at the two indoles (W124, W122), Re(CO)_3_, and dmp along reactive CS1 UKS QM/MM/MD trajectories shown together with absolute values of the indole–indole electronic coupling *H*_ab_ (light blue bars). Letters specify starting ground-state structures (Figure 2). Corresponding spin trajectories are shown in Figure S6.

The temporal evolution of the CS1 electronic structure was revealed by plotting charge (Figures 4 and S5) and spin (Figure S6) at relevant molecular fragments along 3 ps long UKS QM/MM/MD trajectories that started from end-structures of randomly chosen 33 CS1 MM/MD trajectories. Electron density distribution remained essentially stable in 28 cases. Characteristically for CS1, the charges at W124 and W122 were close to +1 and 0, respectively, while a roughly −0.45 charge at dmp is indicative of **Re**^**–**^. Conversion to CS1 was observed in three cases (C, B, E shown in Figure 4). The initially localized CS1 state evolved into a delocalized region (“ET-region”) where the charges as well as spins at the two indoles fluctuated rapidly around 0.5. Some of these fluctuations tended toward localized CS2 or CS1 structures for short time intervals but more often corresponded to delocalized (W124;W122)^•+^ structures. ET-regions lasted for 500–1000 fs, after which electronic structures were converted to CS2 (W124;W122^•+^). However, the CS2 charge/spin distribution appeared to be 10–15% delocalized between the two indoles, in contrast to fully localized CS1. Charge and spin distributions at the Re atom, Re(CO)_3_ fragment, and the dmp^•–^ ligand were not affected by changes in electron distributions at the two indoles.

Two CS1 QM/MM/MD trajectories (A, D in Figures S5 and S6) exhibited dmp^•–^ → W124^•+^ back ET that regenerated *Re in a predominantly dmp-localized ^3^ππ* intraligand (^3^IL) electronic structure. This accords with the experimentally established reversibility of *Re ← W124 ET (equilibrium constant 1.75).^15^

### ET-Enabling Conditions

**Figure 5:**
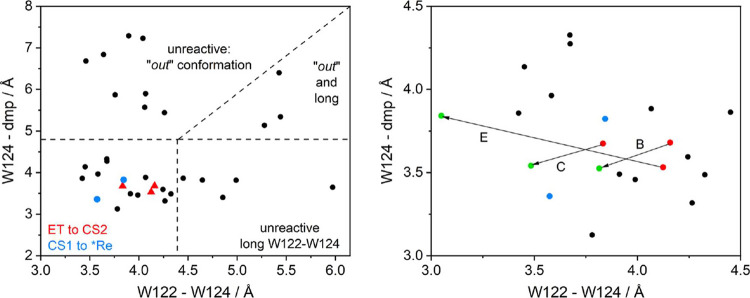
Outcomes of 3 ps-long CS1 QM/MM/MD trajectories as a function of initial geometries of UKS QM/MM/MD simulations. Red: initial geometries of trajectories showing ^3^CS1 → ^3^CS2 conversion (W124^•+^ ← W122 ET). Blue: ^3^CS1 → *Re(^3^IL) (dmp^•–^ → W124^•+^ back-ET). Black: no reaction. Dashed lines show the reactivity regions discussed in the text. Right: detail of the region of reactive trajectories, including geometries at times when the charges at W124 and W122 first equalized (green). Distances are defined, as shown in Figure 2.

**Figure 6:**
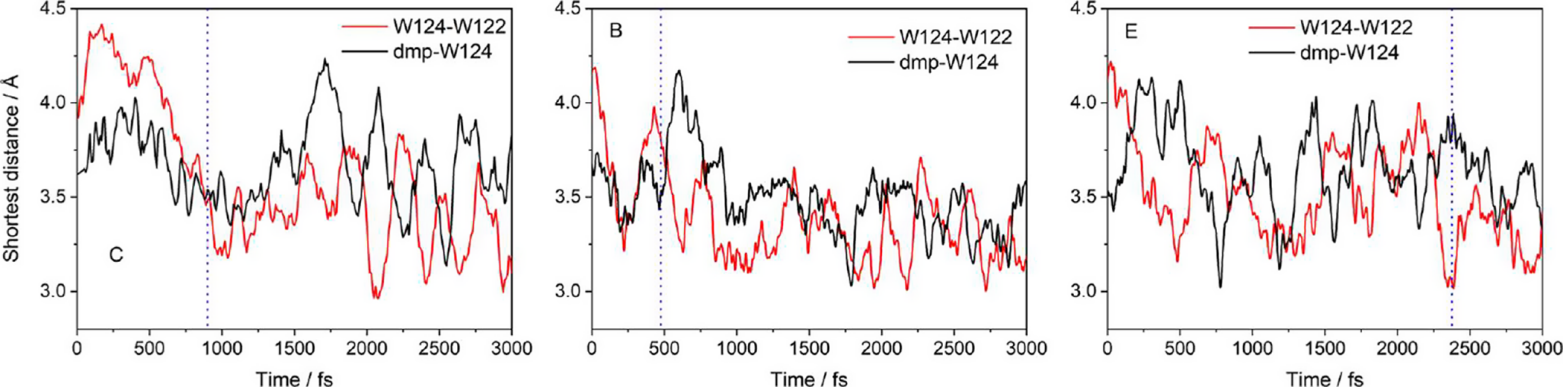
Reactive CS1 QM/MM/MD trajectories of the closest dmp-W124 and indole–indole distances in **Re126W124W122Cu**^**I**^. The dotted vertical line marks the start of the ET region–the first time the W124 and W122 charges were equal (see Figure 4).

The finding that only 3 out of 33 calculated CS1 trajectories exhibited conversion to CS2 confirms that the W124^•+^ ← W122 ET is a low-probability event. Next, we attempted to trace ET-enabling factors by analyzing individual trajectories and relating their outcomes to initial structures and dynamic evolutions. Figure 5 shows the outcomes of all calculated 3 ps QM/MM/MD CS1 trajectories as a function of the starting indole–indole and dmp-W124 distances. The three reactive QM/MM/MD CS1 trajectories (C, B, E) started in a relatively narrow region of GS structures. The dmp-W124 distances fluctuated between 3.0 and 4.2 Å (Figure 6), which is well within the range typical for the ″*in*″ conformation. On the other hand, no ET was observed in any of the 11 QM/MM/MD trajectories that started in the ″*out*″ form (upper half of Figure 5).

The indole–indole distance is another important factor. The three reactive QM/MM/MD CS1 trajectories started at the shortest W124–W122 distance between 3.8 and 4.2 Å that contracted on going to the ET region (Figure 5-right, Figure 6). QM/MM/MD distance trajectories (Figure 6) showed ET commencing either in the course of fast indole–indole distance shortening (C and B) or immediately after a sharp drop (E). The indole–indole distance kept fluctuating in the ET region, occasionally increasing, but never above 3.8 Å. Similar conclusions can be drawn from center-to-center distances that are less affected by fluctuations of intramolecular structures. The indole–indole angle fluctuated around a mean value of 83°, without any apparent relation to ET (Figure S7).

The electronic coupling, *H*_ab_, between CS1 and CS2 (approximated as indole-localized diabatic states) was calculated along QM/MM/MD trajectories by CDFT-CI^25,30^ (Supporting Information, Section S1.5.). *H*_ab_ initially fluctuated between 4 and 30 meV and then rose above 40 meV on approach to the ET region, often exceeding 100 meV (Figure 4). Values of ≥100 meV persisted in the CS2 region, indicating much stronger indole–indole electronic interaction than in CS1. In terms of structural parameters, *H*_ab_ correlates with W124–W122 center-to-center distances (correlation coefficients −0.77 (C), −0.46 (B), −0.70 (E)), whereas no correlation was found with their angles (Figure S7). The large *H*_ab_ values indicated that W124^•+^ ← W122 ET is adiabatic, thereby implying that the ET is controlled by solvent and protein dynamics. (For example, with *H*_ab_ = 40 meV and λ = 800 meV, the Landau–Zener parameter 2πγ = π^3/2^<*H*_ab_^2^>/*h*ν_eff_√(λ*k*_B_*T*) equals 1 for ν_eff_ = 1.5 × 10^13^ s^–1^ (66 fs)^–1^. Protein and solvent motions in Re-labeled azurins are orders of magnitude slower,^31,32^ affording 2πγ ≫ 1, ensuring adiabaticity.^33,34^)

**Figure 7:**
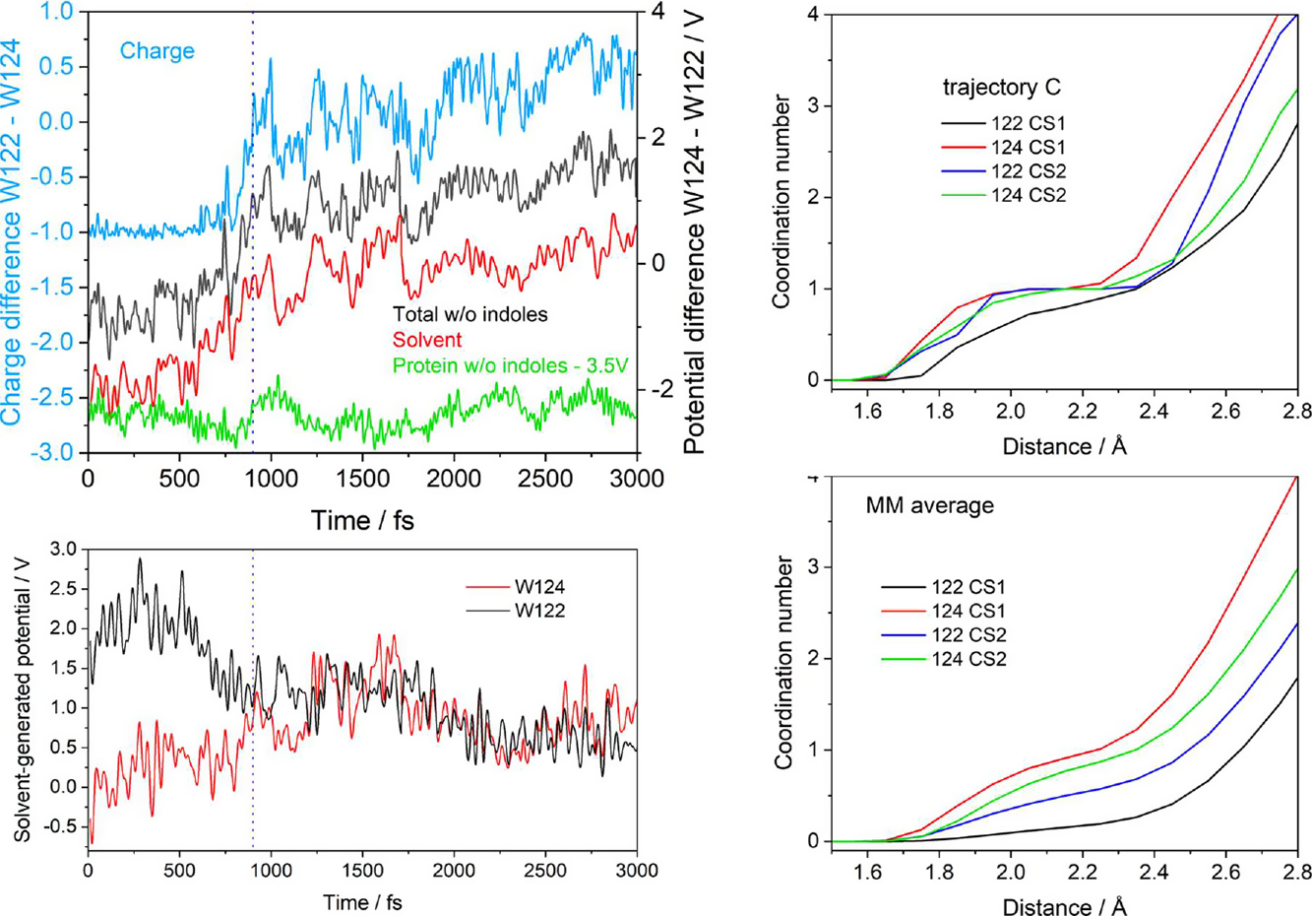
Left: Temporal evolution of charges and potentials along trajectory C. Top: Difference of electrostatic potentials at W124 and W122 generated by all atoms in the system except the other indole (Δφ, black), by the solvent (Δφ(solv), red), and by the protein except the other indole (Δφ(prot), green, shifted by −3.5 V for clarity). The charge difference Δ*q* between W122 and W124 is shown in blue. Bottom-left: Temporal evolution of solvent-generated electrostatic potentials at W124 and W122. (To calculate potentials at W124 except the other indole, W122 was taken out of the system and vice versa. This procedure excluded electrostatic effects of the shifting charge.) For individual potentials at W124 and W122, and the effect of excluding the other indole; see Figure S9. Potential variations due to Na^+^ ions (included in φ(solv)) were negligible. The dotted vertical line marks the start of the ET region, the first time the charges at W124 and W122 were equal. Right: W122 and W124 water coordination numbers calculated from the CS1 and CS2 parts of the reactive trajectory C (top) and averaged over CS1 and CS2 MM/MD trajectories (bottom). Each water molecule was assigned solely to its closest residue. The nonoverlapping proximal volume shells used in the calculations are depicted in Figure S12. Coordination numbers and *g*(*r*) values for all three reactive trajectories are compared in Figures S13 and S14.

Environmental (protein, solvent) dynamics, which affect W124^•+^ ← W122 ET, were analyzed in terms of the temporal evolution of electrostatic potentials generated by surrounding atoms at scaled van der Waals surfaces of the two indoles (depicted in Figure S8, details in the Supporting Information, Section S1.6.). Comparing potentials generated by different parts of the system provided further insight into the origins of the environmental effects: (i) potentials φ(124) and φ(122) were generated by all atoms of the system except the indoles, (ii) potentials generated by the solvent, abbreviated φ(124-solv) and φ(122-solv), (iii) potentials generated by the protein without indoles, φ(124-prot) and φ(122-prot) (protein includes **Re**^**–**^), and (iv) by **Re**^**–**^ separately. Potential trajectories C, B, and E exhibited common patterns leading to ET, which will be demonstrated on trajectory C (Figures 7 and S9; for B and E see Figures S10 and S11). The crucial role of the environment in driving W124^•+^ ← W122 ET was demonstrated by close correlations between differences of the charges and potentials at the two indoles, Δ*q* = *q*(122) – *q*(124) and Δφ = φ(124) – φ(122). The charge and potential differences fluctuated independently of each other until about 560 fs before the ET region, at which point they became strongly correlated. The best correlation was found when potential-difference fluctuations preceded charge-difference changes by a few femtoseconds, indicating that changes in electrostatic potentials at the two indoles drive ET. They remained correlated throughout the ET period and also in the CS2 region. Several trends emerged during the 500–400 fs period before ET onset: Initially, deep in the CS1 region, φ(124) was calculated as 1.5–2 V more negative than φ(122), thereby showing that the environment stabilizes W124^•+^. Then, φ(124) and φ(122) trajectories approached, equalized, and eventually crossed each other before entering the ET region. Δφ increased and became positive before ET, indicating increasing environmental electrostatic stabilization of W122 relative to W124^•+^. The ET region started when Δφ reached a level of about +1.1 V. This behavior originated mainly from a rapid decrease of φ(122). At later times, φ(122) and φ(124) kept slowly decreasing and increasing, respectively, and their divergence stabilized CS2 and localized the hole mostly at W122.

Trends and fluctuations of total (Δφ) and solvent-generated (Δφ(solv)) potential differences mainly copied each other, showing that Δφ variations to a large degree originated from solvation dynamics. Δφ(solv) increased toward 0 V at the ET onset, revealing that equalizing solvation of the two indoles is an important ET-promoting factor. Convergence of φ(124-solv) and φ(122-solv) to approximately the same level at initial ET stages came mainly from a φ(122-solvent) decrease that started a few hundreds of femtoseconds before the ET region. In B and E, φ(122-solv) dropped sharply at the ET onset and a smaller φ(124-solvent) increase contributed by destabilizing W124^•+^. Trends in solvent-generated potentials indicated that water molecules solvating W124^•+^ started shifting toward W122 about 400 fs before the ET region, driving the system toward ET mainly by stabilizing CS2 (W122^•+^), later aided by a minor contribution from CS1 (W124^•+^) destabilization.

This solvation picture was further corroborated by calculating (Supporting Information, Section S1.7.) water proximal distribution functions^14,35,36^*g*(*r*) and coordination numbers (CN) of the two indoles in CS1 and CS2 portions of the reactive QM/MM/MD trajectories and, independently, in CS1 and CS2 states modeled separately by averaging all respective MM/MD trajectories (Figures 7-right, S13, S14). Going from CS1 to CS2, W124 solvation diminished in the second solvation sphere (ca. 2.3–4 Å) that also shifted farther from W124 by 0.2–0.3 Å. At the same time, W122 solvation was enhanced in the first solvation sphere (1.6–2.2 Å), documented by increases in both *g*(*r*) and the coordination number, and by shifting the *g*(*r*) maximum approximately 0.1 Å closer. The W122 second coordination sphere shifted closer to W122 by ≤0.5 Å, behavior that accords with water molecules moving from a broader region around W124^•+^ in CS1 to the immediate vicinity of W122^•+^ in CS2, combined with the contraction of the W122 second solvation sphere. In absolute terms, the number of H_2_O molecules around W124^•+^ in CS1 is larger than that around W122 (based on trajectory C, up to ∼4 Å). In CS2, the coordination number of W122^•+^ is similar to W124 up to ∼2.5 Å and larger between 2.5 and 3.1 Å, which is in the contracted W122 second solvation sphere region. On the other hand, neutral W122 in CS1 is less solvated than neutral W124 in CS2. The same is true for W122^•+^ in CS2 and W124^•+^ in CS1 (Figure 7 right-bottom). The generally weaker solvation of W122 than W124 (either neutral or cationic) is attributable to W122 shielding by the S118A119L120 α-helix segment (further referred to as SAL), whose A119 oxygen atom is within an H-bonding distance of the W122 indole-NH group.^14^

**Figure 8:**
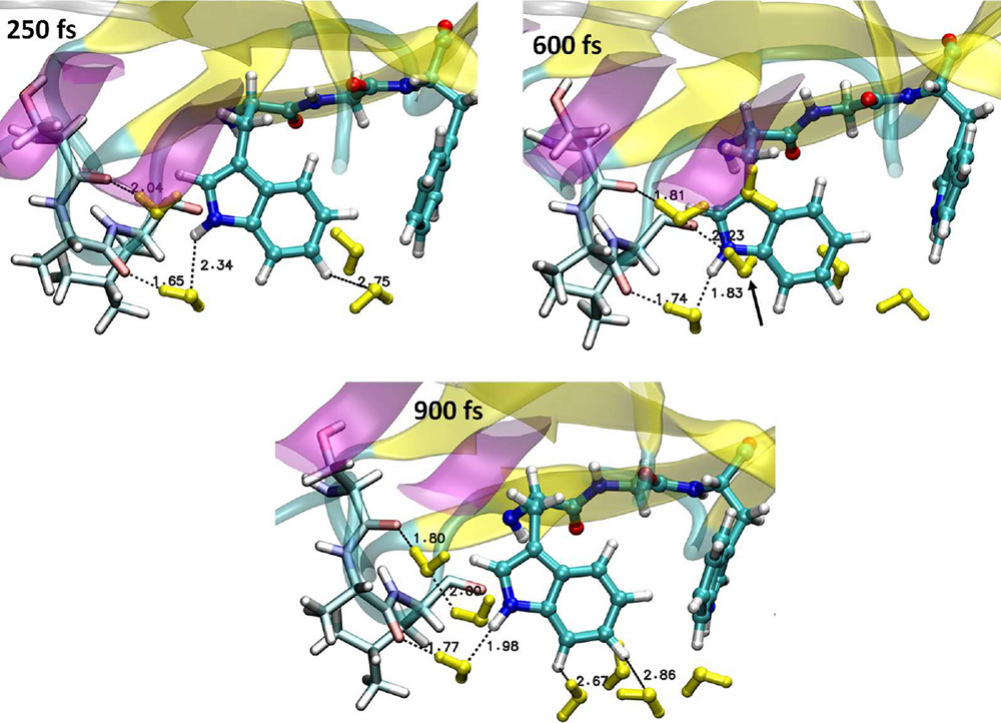
Water molecules within 3.2 Å from the W122 indole at three snapshots of trajectory C. At 250 fs, the system is in the localized CS1 state. At 600 fs, the system evolves toward the ET region; 900 fs is the time when the charges at W124 and W122 become equal for the first time. The A119-O···H_2_O···HN(W122) hydrogen-bonded bridge is absent at 250 fs, forms by ∼500 fs, and then stays through the ET and CS2 regions (Figure S17). Another H_2_O molecule appears 3.00 Å from HN(W122) at 600 fs (arrow) and remains close through the beginning of the ET region. Solvation of the W122 aromatic ring increases between 600 and 900 fs, as water molecules in the second solvation sphere shift from W124 toward W122.

Although Δφ and Δφ(solv) trajectories largely copied each other, they were not exactly parallel, owing to varying contributions to Δφ from the protein potential (Figures 7, S15). Differences between φ(124-prot) and φ(122-prot) showed modest variations along reactive trajectories (green curves in Figures 7, S10 and S11). The ET onset occurred in a shallow Δφ(prot) minimum, owing in part to potential changes generated by the SAL segment (Figure S15) that moved away from the nearby W122 before and/or at the beginning of the ET region (Figure S16). Increasing φ(122-prot) alone would destabilize CS2 and hinder ET. However, the overall effect was the opposite: the spatial opening between W122 and SAL, together with a less negative potential from the protein, allowed a water molecule to squeeze in and form a stable H-bond between the W122 indole-NH and the A119 O atom ca. 400 fs before the start of the ET region (Figure S17). At the same time, another water molecule moved in to form a S118 amide-O···H_2_O···H_2_O···HN-W122 H-bonded chain and another water molecule moved close to the W122 indole-NH from around W124^•+^. Solvation of the W122 aromatic rings increased at later stages before ET. (Increasing W122 solvation is visualized in Figure 8, and water dynamics are shown in Figure S17.) H-bonding to the W122 indole-NH in combination with a water shift from W124^•+^ toward W122 in the second solvation sphere led to the drop of φ(122-solv) and a modest rise of φ(124-solv) on approach to the ET region. Solvation changes that drive ET thus appear to be triggered by coupled protein and water dynamics, namely, by the relative motion of W122 and the SAL segment whereby protein dynamics help to equalize W124 and W122 solvation before the ET region. In addition, a small increase in the level of φ(124-prot) aided ET by destabilizing CS1. It originated mainly from changes in the **Re**^**–**^-generated potential (Figure S18) that became more positive by virtue of ca. +0.1 e charges on H atoms of dmp CH_3_ groups. Later in the ET and CS2 regions, a larger increase in φ(124-prot) than φ(122-prot) led to the W122 and W124 potential trajectories crossing each other, while trajectories of solvent-generated potentials remained comparable and, in the CS2 region, helped to localize the hole predominantly at W122 in the CS2 state.

### ET-Disabling Conditions

Figure 5 contains 28 unreactive trajectories that did not show any ET within the 3 ps QM/MM/MD simulation time. Based on their positions in the indole–indole/dmp-W124 distance space, they can be sorted into three groups:

First, those with long indole–indole distances (>4.2 Å) were deemed unreactive because of weak electronic coupling. At those distances, ET would be slow (nonadiabatic). Also, the two distant indoles are solvated essentially independently of each other, not allowing for W122^•+^ stabilization by solvation dynamics.

The second set of 7 unreactive trajectories started in the same region as the reactive ones or at somewhat shorter indole–indole distances (≤3.8 Å). The lack of reactivity cannot be attributed to weak electronic coupling. The average *H*_ab_ over such a typical trajectory was calculated as 10 meV and values of tens of meV (up to 55 meV) were reached in the regions of short indole–indole distances (Figure S19). These *H*_ab_ values are comparable to those found at the ET onset in the reactive trajectories, but no state crossing was observed. We suggest that this behavior is attributable to insufficient solvation of W122 at short distances that in turn is caused by the proximity of the SAL segment (Figure S19). (The average SAL-W122 shortest distances were calculated to be 0.7 (S118), 1.5 (A119), and 0.3 Å (L120) shorter than at the ET onset of the reactive trajectory C.) Accordingly, water *g*(*r*) and coordination numbers (calculated at any distance shorter than 2.5 Å) were comparable to those obtained from reactive trajectories (e.g., CN = 1.06 at 2.25 Å) for W124 but much smaller for W122 (0.24 for the unreactive case vs 0.8 for trajectory C), see Figure S19. Limited W122 solvation produced unfavorable relative electrostatic potentials at W124 and W122: φ(124) and φ(122) trajectories did not cross (their difference stayed around −0.5 V, well below the +1.1 V threshold inferred from reactive trajectories). φ(124-solv) stayed below the φ(122-solv) trajectory by 1–2 V, failing to meet another ET-enabling condition of equalizing solvent-generated potentials (Figure S20). Also, the **Re**^**–**^ - generated potential at W124^•+^ was ca. 0.2 V lower than at the reactive trajectory C and did not show positive fluctuations that would destabilize W124^•+^ and help trigger ET. Apparently, the protein and solvation dynamics did not act in concert to stabilize CS2 and destabilize CS1, keeping the two states energetically apart, at least during the 3 ps simulation time.

The third group consisted of 8 unreactive trajectories in the upper-left quadrant of Figure 5. These trajectories all started at favorably short indole–indole distances, but **Re**^**–**^ was in the ″*out*″ conformation where dmp and the axial CO pointed toward the solution, with one equatorial CO ligand toward W124^•+^, and the second toward another β-sheet, namely, the A19I20T21 segment. The absence of reactive 3 ps CS1 QM/MM/MD trajectories suggested that **Re**^**–**^ rotation away from W124^•+^ hinders the ET process. (A single simulation run for 9 ps did not show ET either.) This conclusion raised two questions, namely, what drives **Re**^**–**^ rotation in the CS1 state and why does the probability of W124^•+^ ← W122 ET depend on the orientation of the dmp^•–^ ligand relative to W124^•+^.

**Re**^**–**^ rotation rapidly depleted the initial ″*in*″ CS1 population. The number of ″*in″* structures in CS1 MM/MD trajectories fell to about 30% during the first 1 ns. The preference for the ″*out*″ form in CS1 came mainly from larger electrostatic stabilization of W124^•+^ owing to a lower potential generated by **Re**^**–**^ (namely one of the CH_3_ groups of dmp^•–^ and one of the equatorial CO ligands). A smaller additional effect came from the Q107 residue of a neighboring β-sheet whose side chain partly moved into the void between **Re**^**–**^ and W124^•+^ and shortened the distance between W124 and the O atom of the Q107 terminal amide. (Electrostatic ″*out*″ stabilization is documented in Figures S21 and S22 by partitioning the total potential to components generated by solvent, protein, **Re**^**–**^, and Q107. Structures are compared in Figure S23.) In addition, the Re(CO)_3_^δ+^ moiety is stabilized by the potential exerted by the rest of the system. By averaging electrostatic contributions over several QM/MM/MD ″*in*″ and ″*out*″ trajectories, we estimated the ″*out*″ electrostatic stabilization of the cofactors relative to ″*in*″ as −0.18 eV (obtained as a sum of potential × charge terms of relevant fragments).

The absence of W124^•+^ ← W122 ET in ″*out*″ QM/MM/MD trajectories is attributable to the same factors outlined above for unreactive ″*in*″ conformations, only more pronounced (compare Figures S19 and S24). For ″*out*″, W124^•+^ in CS1 was even more electrostatically stabilized, keeping CS1 and CS2 energetically farther apart and increasing the W124^•+^ ← W122 ET energy barrier by about 0.18 eV relative to that of the ″*in*″ conformer (assuming that the transition-state energy does not depend on the conformation). The average Δφ value was 0.12 V more negative than for the unreactive ″*in*″ conformations (Figure S21), pushing the Δφ trajectory even deeper below the +1.1 V threshold. Also, it is likely that the small number of water molecules in the vicinity of W122 (Figure S24) would not be sufficient to support hole transfer to W122.

### CS2 Dynamics and Reverse ET

**Figure 9:**
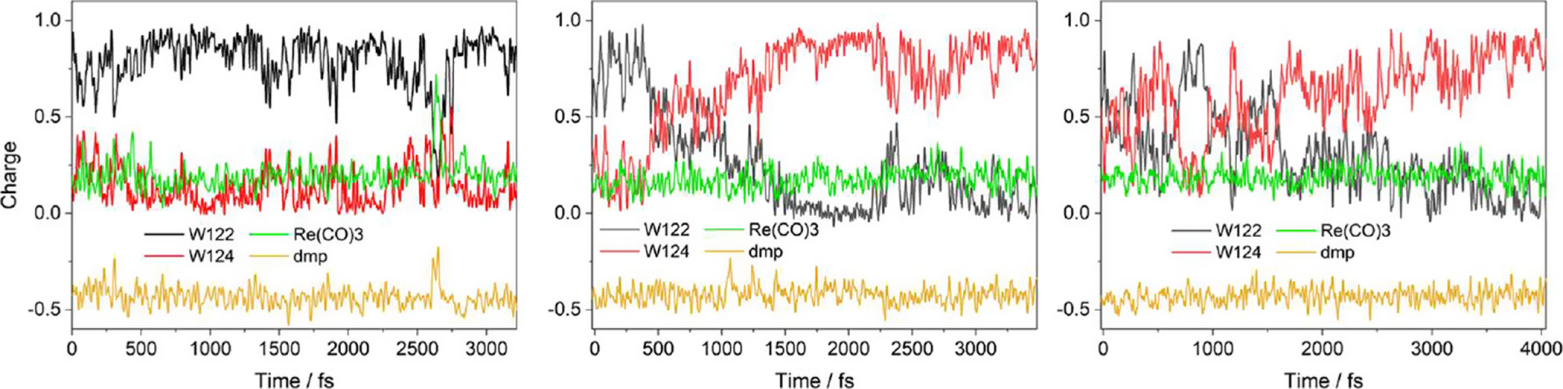
Typical CS2 QM/MM/MD charge trajectories calculated starting from structures generated by 1 ns MM/MD CS2 runs. Left: stable CS2. Middle: reverse ET to CS1 occurs after ∼480 fs. Right: Reversible multiple switching between CS2 and CS1, followed by slow CS1 relaxation, tends toward full hole localization at W124.

The reactive trajectories C, B, E revealed that CS2 is more delocalized than CS1: the hole at W122 was about 85–90% delocalized, while 15–10% remained at W124 (Figure 4). Still, CS2 is structurally and dynamically distinct, in accord with experimental kinetics.^15^ A similar conclusion can be drawn from independent QM/MM/MD simulations of CS2 preprepared by 1 ns MM/MD (Figure 9). Partial electronic delocalization of CS2 appears to be enabled by the environment, since the difference between φ(124) and φ(122) was calculated to be much smaller than for CS1 and Δφ(solv) close to 0 V indicated nearly equal solvation of the two indoles. Δφ also was either close to 0 V or slightly positive. Limited environmental stabilization of W122^•+^ can be attributed to shielding by the SAL segment.

The 11 calculated QM/MM/MD CS2 trajectories exhibited three types of behavior (Figure 9): Reverse W124 → W122^•+^ ET to CS1 (3 cases), back-and-forth ET between the two indoles, i.e., CS1/CS2 switching (4×), and a stable CS2 electronic structure (4×). *H*_ab_ wildly fluctuates (29 meV mean), reaching values around 80 meV (122 meV maximum). This behavior accords with the experimentally observed equilibrium between CS1 and CS2, which is shifted toward CS1 (*K*_2_ = 0.65).^15^ It is of interest that CS1 formed by reverse ET from CS1 is partly delocalized (Figure 4 – left), unlike CS1 produced in the first hopping step, *Re ← W124. Apparently, structural relaxation and the resulting hole localization on W124 take longer than the 3 ps simulation time.

## Discussion

Dynamics simulations afforded a model of photoinduced hole hopping in **Re126W124W122Cu**^**I**^ that qualitatively accounts for experimental observations (Scheme 1).^15^ Simulations also shed light on the factors that control the hopping process, including the structures of the intermediate states: Optical excitation of **Re** produces *Re, a mixed ^3^MLCT/IL state, which oxidizes W124 in multiexponential (≤500 ps) adiabatic ET through a close through-space dmp-W124 contact. It is largely controlled by solvation dynamics, shifting water molecules near W124.^14^ The CS1 state Re^I^(H126)(CO)_3_(dmp^•–^)(W124^•+^)(W122)Cu^I^ is formed in the ″*in*″ conformation, where dmp^•–^ is positioned close to W124^•+^. CS1 then either converts to CS2 by W124^•+^ ← W122 ET or Re^I^(CO)_3_(dmp^•–^) rotates around the Re-His126 bond to the ″*out*″ conformation, rendering CS1 unreactive on the time scales examined here.

W124^•+^ ← W122 ET is adiabatic, driven by the coupled dynamics of the protein and water environments around W124···W122. The system starts to evolve toward ET when W122 moves away from the shielding SAL protein segment and toward W124^•+^. Simultaneously decreasing the W122–W124 distance increases electronic coupling, which accentuates adiabaticity (i.e., environmentally induced dynamic control). At the same time, protein structural fluctuations make W122 more accessible sterically. Water responds by shifting toward W122, which stabilizes CS2 (Figure 8). In particular, two water molecules slip into a growing opening between the SAL and W122. A tight H-bonded bridge forms among the W122 indole-NH group, a water molecule, and the A119 O atom. A second, albeit longer, H-bonded bridge connects NH with the S118 amide O atom. A shift in the second solvation sphere follows, which further stabilizes CS2 and somewhat destabilizes CS1. This shift lasts for several hundreds of femtoseconds, during which the CS2 and CS1 energies approach each other, owing to decreasing (W122) and slightly increasing (W124^•+^) electrostatic potentials generated by the environment (mainly water) at the two indoles. Fluctuations of differences in the electrostatic potentials of the two indoles trigger charge redistribution. At later stages, subtle protein structural changes increase the electrostatic potential exerted by **Re**^**–**^ at W124^•+^, which in turn further destabilizes CS1. Together, these environmental dynamics drive the CS1 and CS2 energies closer, and they eventually become equal. At that point, the hole is delocalized between the two indoles, and the system enters an ET region that lasts for up to 1 ps. Here, the structure and charge localization fluctuate (Figure 4). Differences between electrostatic potentials at W124 and W122 slightly increase, while the water-derived potentials remain comparable. The system dynamics retain momentum and eventually lead to the CS2 state Re^I^(H126)(CO)_3_(dmp^•–^)(W124)(W122^•+^)Cu^I^, where the hole is partially (10–15%) delocalized between the two indoles. Electrostatic potentials are only slightly more negative at W122^•+^ than at W124. Environment polarization is not sufficient to localize the hole fully at W122, which, together with relatively large coupling, accounts for partial CS2 electronic delocalization.

It is of interest that reactive and unreactive ″*in*″ CS1 trajectories at early stages are very similar as concerns indole–indole distances and orientations, solvation, electrostatic potentials, and coupling. The reactive trajectories begin to differ 500–400 fs before the ET region, owing to the “right” coincidence of structural and solvational conditions and also their simultaneous dynamical evolution toward ET, which is triggered by relatively minor protein changes in the W122 vicinity. Searching the structural and solvational space for low-probability ET-promoting conditions is responsible for the relatively slow W124^•+^ ← W122 ET rate.

W124^•+^ ← W122 ET (CS1/CS2 conversion) is the *Re ← W124 ← W122 ← Cu^I^ hopping bottleneck. The low yield of Cu^I^ oxidation was attributed to a shift in CS1 ↔ CS2 equilibrium to the left (*K*_2_ ≅ 0.65) in combination with competitive (30–60 ns) charge recombination **Re**^**–**^/W124^•+^ to the ground state (Scheme 1).^15^ In accordance with that proposal, a majority of CS2 trajectories either returned to CS1 or showed frequent CS1/CS2 switching; and only a few led to a stable CS2 state. Facile ET back to CS1 is understandable in view of the calculated electrostatic potentials at the two indoles, which are much closer to each other in CS2 than in CS1 and with almost equal solvent-generated components. Small structural/solvent fluctuations in the opposite direction could return the hole back to W124, especially since CS2 is partly delocalized and the two states remain strongly coupled.

Simulations revealed rotation of Re(CO)_3_(dmp) relative to W124 as an additional mechanism that diminishes the overall hole-hopping quantum yield in two ways: Ground-state **Re126W124W122Cu**^**I**^ occurs as a ca. 3:2 mixture of ″*in*″ and ″*out*″ conformers (Figure 1a,b) that frequently convert between each other. Near-UV irradiation excites both forms to *Re but only ″*in*″-*Re reacts further, to ″*in*″-CS1. Hence, about 40% of absorbed photons do not drive hopping. The electronically excited complex *Re is conformationally stable, and conversion to ″*out*″ causes only small losses (Figure 3). On the other hand, the ″*in*″-CS1 population is depleted by **Re**^**–**^ rotation to ″*out*″-CS1, in competition with its conversion to CS2 (Figure S25). The ″*out*″-CS1 conformer is much less reactive and its presence likely is responsible for the slow (∼8 ns) total rate of W124^•+^ ← W122 ET. Moreover, both ″*in*″ and ″*out*″ CS1 conformers undergo experimentally established^15^ 30–60 ns **Re**^**–**^ → W124^•+^ back-ET to the GS (Scheme 1). This deactivation step competes with forward ET, more so for ″*out*″-CS1, owing to its slow forward-ET rate. The CS1 conformational change thus emerged from simulations as a previously unrecognized competitive side reaction that slows the hopping process and diminishes its quantum yield. It would be virtually impossible to distinguish the ″*in*″ and ″*out*″ forms spectroscopically since the same **Re**^**–**^ infrared and visible chromophore is simultaneously present in three different forms: ″*in*″ and ″*out*″ CS1, and the RP (Scheme 1). The small blue shift observed^15,37^ in the TRIR spectrum is most likely caused by the conversion to CS2 and/or RP. On the other hand, a shoulder observed^38^ at the lowest ν(C≡O) IR band of **{Re126T124W122Cu**^**I**^**}**_**2**_ in an intermolecular (**Re**^**–**^)(W122^•+^)’ CS state likely is attributable to an analogous ″*out*″ form.

The loss of W124^•+^ ← W122 ET reactivity upon **Re**^**–**^ rotation to the “out” position shows that **Re**^**–**^ is more than a spectator to subsequent charge hopping. **Re**^**–**^, W124^•+^, and W122 share the second solvation layer and **Re**^**–**^ rotation is accompanied by changing water coordination numbers that decrease at W122 and increase at W124^•+^, which is exactly opposite to changes that would drive W124^•+^ ← W122 ET toward completion. The CS1-stabilizing decrease in the electrostatic potential at W124^•+^ upon rotating dmp^•–^ away is counterintuitive. The **Re**^**–**^-generated potential at W124^•+^ in the ″*in*″ form does not arise from the negative charge delocalized over dmp^•–^ but rather from the partial positive charge at CH_3_ H atoms that move away upon dmp^•–^ rotation. Also of interest is that small protein structural changes accompanying conformational fluctuations decrease the potential at W124^•+^ by moving Q107 (of a different β-sheet) closer and reorienting its side chain so that the terminal −C(NH_2_)=O group points toward W124^•+^ (Figure S23). All these changes, which are important for functional hopping, would be very hard to predict without simulations.

Adiabaticity of the W124^•+^ ← W122 step implies that environmental dynamics drive CS1 and CS2 states to degeneracy and carry the system over the energy barrier. The simulations did not, however, provide direct information about the energy barrier itself. Considering Δ*G* ≅ +11 meV and a reorganization energy (λ) of 800 meV, Δ*G*^#^ of the W124^•+^ ← W122 step is estimated to be 210 meV, which is lowered owing to large coupling in the crossing region to ca. 170 meV (assuming *H*_ab_ of 40 meV at the state crossing, *H*_ab_’ around the CS1 minimum of 10 meV (Figure 4) and using a correction factor^34^*H*_ab_ – (*H*_ab_’)^2^/λ). This barrier leads to an adiabatic reaction rate of ν_N_ × 1.2 × 10^–3^ s^–1^, where ν_N_ is the effective frequency of motion along the reaction coordinate. Dynamic phosphorescence and infrared absorption Stokes shift studies of various Re-labeled azurins revealed multiexponential solvation and protein relaxation dynamics ranging from picoseconds to tens of nanoseconds (plus a low-amplitude μs component).^31,32^ Although not all these motions need to be coupled to ET, ν_N_ can be safely estimated to be less than (100 ps)^−1^, predicting ET to be slower than 83 ns, contrary to experiment (ca. 8 ns, likely faster for ″*in*″-CS1). Fast ET rates, short contacts, strong through-space coupling between aromatic side chains, and complex relaxation dynamics covering a broad temporal range make **Re126W124W122Cu**^**I**^ similar to systems for which a nonequilibrium short-range ET mechanism^33,39−41^ has been postulated, as in Trp-containing flavoproteins.^39,42−45^ This model is applicable to ET coupled to environmental (solvent) relaxation dynamics occurring on a comparable time scale. Coupling with environmental fluctuations reduces the outer sphere reorganization energy λ_o_ and the driving force but by different amounts.^33,39,40,46^ It accounts well for experimentally determined ultrafast rates and predicts stretched-exponential kinetics.^39^ In accord with prediction, the first hopping step in all Trp-containing Re-azurins investigated so far displays multiexponential kinetics.^13,15,37,38,47^ Such behavior likely occurs also for the second “hop” W124^•+^ ← W122, although detailed kinetics information is not yet available.

**Re126W124W122Cu**^**I**^ can be viewed as an artificial counterpart of natural systems undergoing fast photoinduced ET between an excited chromophore and an aromatic amino acid, as in flavoproteins,^39,42−45^ GFP-family of proteins,^48^ and, especially, photolyases (PL) and cryptochromes (CRY) that have been extensively studied both experimentally^6−8,49−53^ and theoretically.^46,54−57^

Comparing **Re126W124W122Cu**^**I**^ with the evolution-conserved and, presumably, optimized hole-hopping systems in the PL and CRY families is especially instructive. In PL, a hole is carried from a photoexcited chromophore (flavin radical FADH^•^ in the resting state^6,7,49^ or FAD in the oxidized form^8^) to the protein surface through a chain of three (in some cases four^51,56^) tryptophans. The hole transport in PL is completed in about 100 ps, and the terminal, surface-exposed tryptophan is oxidized with a quantum yield of 0.19 (measured for the FADH^•^ state^7^) or 0.4 (estimated from kinetics for the FAD state^8^). Hopping is unidirectional as all forward ET steps are much faster than the corresponding back reactions and selective, whereby the productive pathway is followed even in the presence of other close-lying Trp residues.^8,49,54^

Compared with PL, hole transfer in **Re126W124W122Cu**^**I**^ is much slower (5–8 ns^15^ vs 2.5–150 ps^49^) and less efficient. In either PL or CRY, tryptophan residues along the hopping chain are differentiated by unique protein and solvation environments that affect their respective formal potentials and, hence, driving forces of hopping steps. The main difference between the two systems lies in the cofactor solvation: In PL/CRY, hole hopping proceeds from the protein interior toward the surface through Trp residues that are progressively more water-exposed.^46,55,56^ Increasing solvation and water orientational polarization stabilizes Trp^·+^ and lowers the next Trp^·+^/Trp formal oxidation potential relative to the preceding one. (For example, potentials of the three Trp residues in *E. coli* PL decrease going from the one next to FADH^•^ to the surface: 1.61, 1.45, and 1.25 V vs. NHE.^49^) Hopping thus occurs along an increasing driving-force gradient (Δ*G* ≅ −160 and −200 meV). In contrast, the entire hopping system in **Re126W124W122Cu**^**I**^ is located at the protein surface, and both tryptophans are in contact with water molecules in the aqueous environment. However, the second hopping intermediate (W122) is solvated less than the first one (W124), owing to partial shielding by the SAL segment. Hence, solvation and driving-force gradients are opposite to those in PL/CRY as well as those required for efficient and directional (irreversible) ET. W124^•+^ ← W122 ET is uphill (Δ*G* ≅ +11 meV)^15^ and the equilibrium is shifted to the left, that is, toward CS1. Since electron tunneling from Cu^I^ to W122^•+^ is relatively slow (45–75 ns),^15^ the CS2 state behaves as a “hole reservoir” for the reverse reaction to CS1, from which fast **Re**^**–**^ rotation to the ″*out*″ form and charge recombination to the GS occur, diminishing the quantum yield. Recombination is spin-forbidden (^3^CS1 → ^1^GS) and, as such, slow (30–60 ns). The overall hopping quantum yield in FMNH^•^*E. coli* PL is limited to ∼0.19,^7^ but owing to different reasons: ultrafast (4 ps) spin-allowed recombination between FMNH^–^ and the proximal W^·+^ and relatively short (80 ps) *FMNH^•^ lifetime.^7,49^ The latter is not an issue for **Re126W124W122Cu**^**I**^, where the inherent *Re lifetime is about 1 μs.

Hole-hopping kinetics between Trp residues is surprisingly dependent on the chromophore, even if it does not directly participate in ET. **Re**^**–**^ rotation in CS1 slows down W124^•+^ ← W122 ET, while rates of analogous steps in PL strongly depend on the flavin oxidation state (FMN vs FMNH^•^).^8,49^ Long-range effects such as from the electrostatic field generated by the reduced chromophore and subtle alterations of the protein fold (Q107 and SAL movements in the present case) likely are responsible.

Improving the efficiency of the **Re126W124W122Cu**^**I**^ hopping system (development of a photoenzyme) would require decreasing the electrostatic potential at W122. Modifying the SAL segment could be a way forward. Extending and making it more flexible to enhance W122 water access could be considered, although the effect on the protein-fold stability might be an issue. Alternatively, replacing one of the residues in SAL with a negatively charged/highly solvated residue (aspartate or glutamate) could be a possibility. Restricting **Re** rotation would increase the yield substantially but any chromophore modification would likely affect the first hopping step, *Re ← W124. For the design of artificial photoenzymes employing hopping, it appears that any unnatural photosensitizer (metal complex, nanoparticle, etc.) would have to be attached to a protein surface. Hopping would then occur along the surface, encountering similar problems to those discussed for Re-azurins, which are, in principle, solvable. Recently reported unnatural photoenzymatic processes employed charge-transfer interactions between a naturally occurring flavin chromophore and a reactant in the protein interior, a mechanism that does not involve net electron hopping to or from an active site.^58,59^

Hopping thermodynamics in **Re126W124W122Cu**^**I**^ as well as in PL/CRY are controlled by the electrostatic potential generated by the solvent, which in turn tunes the tryptophan-site energies. This redox tuning most likely operates also in Trp/Tyr chains that protect enzymes against oxidative damage when the natural substrate is not activated for reaction.^1,10^ Examining solvent access to individual Trp/Trp hopping intermediates and simulating environmentally generated electrostatic potentials could be revealing for these hole-hopping processes. It is of interest that estimated coupling values along a Trp/Tyr hopping chain in the mitochondrial cholesterol-metabolizing enzyme *cyt* P450 CYP11A1 vary between 1 and 60 meV,^60^ suggesting alternating tunneling and adiabatic steps.

## Conclusions

The W124^•+^ ← W122 ET step of the **Re126W124W122Cu**^**I**^ hopping system occurs as an adiabatic process controlled by solvent dynamics, aided by subtle protein motions. Lower solvation and partial protein shielding of W122 increase its formal oxidation potential, making ET slightly endergonic. W124^•+^ ← W122 ET is considerably slowed by Re^I^(CO)_3_(dmp^•–^) rotation that re-orients the dmp^•–^ ligand away from W124^•+^ and the protein. Competition between this conformational change and the forward ET step in the CS1 state diminishes the overall hopping quantum yield. Thermodynamics and kinetics of hole hopping between tryptophan residues in Re-azurin systems as well as in natural (photo)enzymes such as photolyases and cryptochromes are determined by electrostatic potentials at individual residues generated by solvating water with smaller contributions from the polypeptide. Electrostatic potentials distinguish individual Trp sites and their fluctuations help carry reacting systems over energy barriers. In natural evolution-optimized hopping systems, electrostatic potentials create favorable redox-potential gradients along the hopping chains. Such redox tuning is difficult to obtain in artificial systems, where hopping often occurs on protein surfaces.

## Supporting Information Available

Trajectories of structural parameters of the ground, CS1, and CS2 states, charge, spin, and electrostatic potentials, water proximal distribution functions, visualizations of typical structures, and characteristics (electrostatic potentials, *g*(*r*), charge distributions) of the ″*in*″ and ″*out*″ conformers, and detailed description of the computational procedure (PDF)
