# Supplementary material for: Tryptophan to Tryptophan Hole Hopping in an Azurin Construct
Source: J Phys Chem B. 2023 Dec 25;128(1):96–108. doi: 10.1021/acs.jpcb.3c06568 (PMC10788906; doi:10.1021/acs.jpcb.3c06568)
Supplement: Supplementary file 1 — jp3c06568_si_001.pdf [file jp3c06568_si_001.pdf]

SUPPORTING INFORMATION

Tryptophan to Tryptophan Hole Hopping in an Azurin Construct

Martin Melčák,[a,b] Filip Šebesta,[a,c] Jan Heyda,[a,b] Harry B. Gray,*[,d] Stanislav Záliš,*[,a] Antonín Vlček*[,a,e]

[a] J. Heyrovský Institute of Physical Chemistry, Czech Academy of Sciences, Dolejškova 3, CZ-182 23 Prague, Czech Republic

[b] Department of Physical Chemistry, University of Chemistry and Technology Prague, Technická 5, CZ-166 28 Prague, Czech Republic

[c] Department of Chemical Physics and Optics, Faculty of Mathematics and Physics, Charles University, Ke Karlovu 3, CZ-121 16 Prague, Czech Republic

[d] Beckman Institute, California Institute of Technology, Pasadena, California 91125, United States

[e] Department of Chemistry, Queen Mary University of London, E1 4NS London, U.K.

Contents

Fig. S1-2:	Ground-state MM/MD trajectories.	S3-4
Fig. S3:	Typical Re126W124W122Cu^{I} structures	S5
Fig. S4:	Unreactive CS1 MM/MD trajectories	S6
Fig. S5-6:	Charge and spin QM/MM/MD trajectories	S6-7
Fig. S7:	Distances and angles along QM/MM/MD reactive trajectories	S8
Fig. S8:	Visualization of an indole surface for calculating electrostatic potentials	S8
Fig. S9-11:	Trajectories of electrostatic potentials	S9-11
Fig. S12:	Visualization of proximal volume shells used to calculate water proximal distribution functions	S12
Fig. S13-14:	Water proximal radial distribution functions	S13-14
Fig. S15:	Trajectories of protein-generated electrostatic potentials	S15
Fig. S16:	Trajectories of W122-SAL distances	S16
Fig. S17:	Evolution of the W122 indole-NH – OH_2 distances	S17
Fig. S18:	Trajectories of **Re**$^{-}$ -generated electrostatic potentials	S17
Fig. S19:	Characteristics of a typical unreactive "*in*" trajectory	S18
Fig. S20:	Electrostatic potentials along a typical unreactive "*in*" trajectory	S19
Fig. S21-22:	Distributions of electrostatic potentials over unreactive "*in*" and "*out*" trajectories	S20-21
Fig. S23:	Structures showing W124, **Re**$^{-}$, and Q107 orientations in reactive and unreactive trajectories	S22
Fig. S24:	Characteristics of a typical "*out*" trajectory	S23
Fig. S25:	Temporal evolution of "*in*"-MLCT and "*in*"-CS1 populations	S24
Section S1.	Computational details	S25
S1.1.	General procedure	S25
S1.2.	Classical MM/MD simulations	S25
S1.3.	QM/MM/MD simulations	S26
S1.4.	CAM-B3LYP test calculations	S27
S1.5.	Electronic coupling	S27
S1.6.	Electrostatic potentials	S28
S1.7.	Proximal volume, coordination number, and distribution function	S29
S1.8.	Atomic charges for the CS1 and CS2 states	S30
References		S35

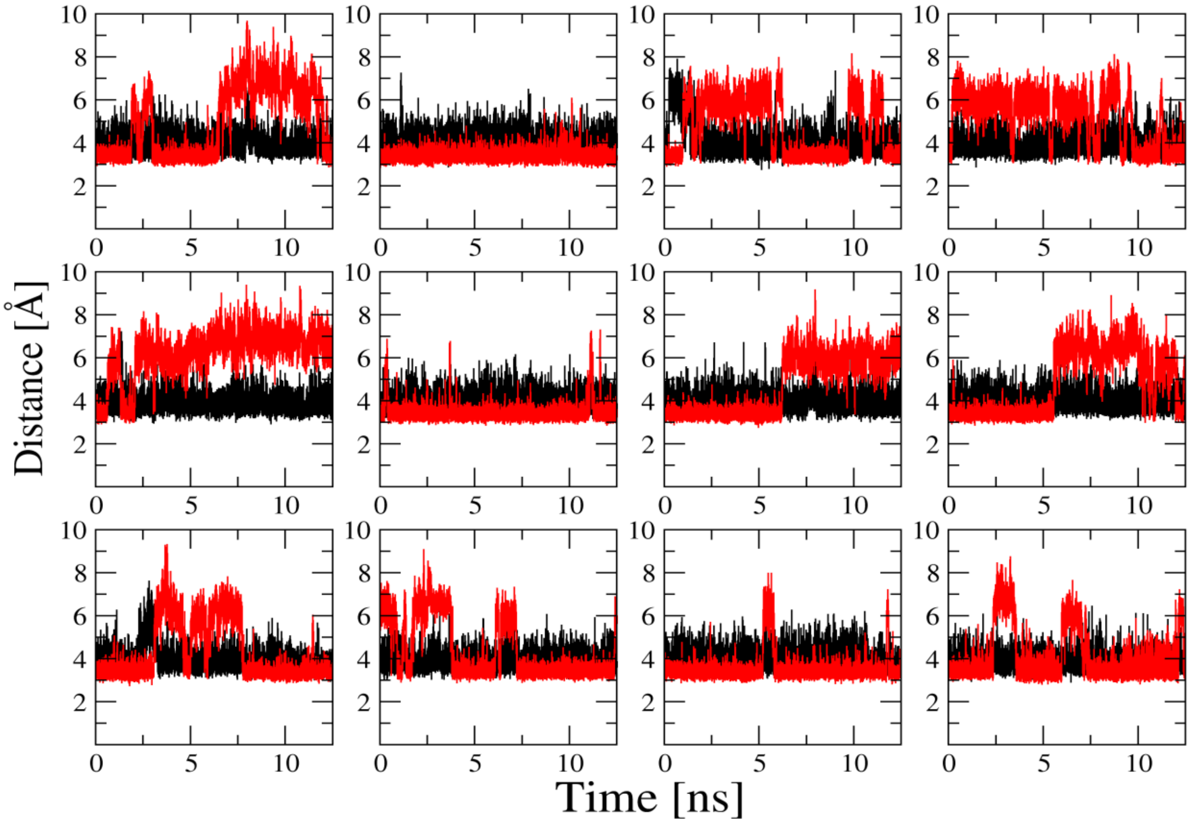

Figure S1. Ground-state MM/MD trajectories of **Re126W124W122Cu^{I}**. Black: the shortest distance between atoms of W122 and W124 indole aromatic rings. Red: The shortest distance between atoms of W124 and dmp aromatic rings. Starting structures for CS1 and CS2 simulations were taken from the first three trajectories, as shown in Figure 2.

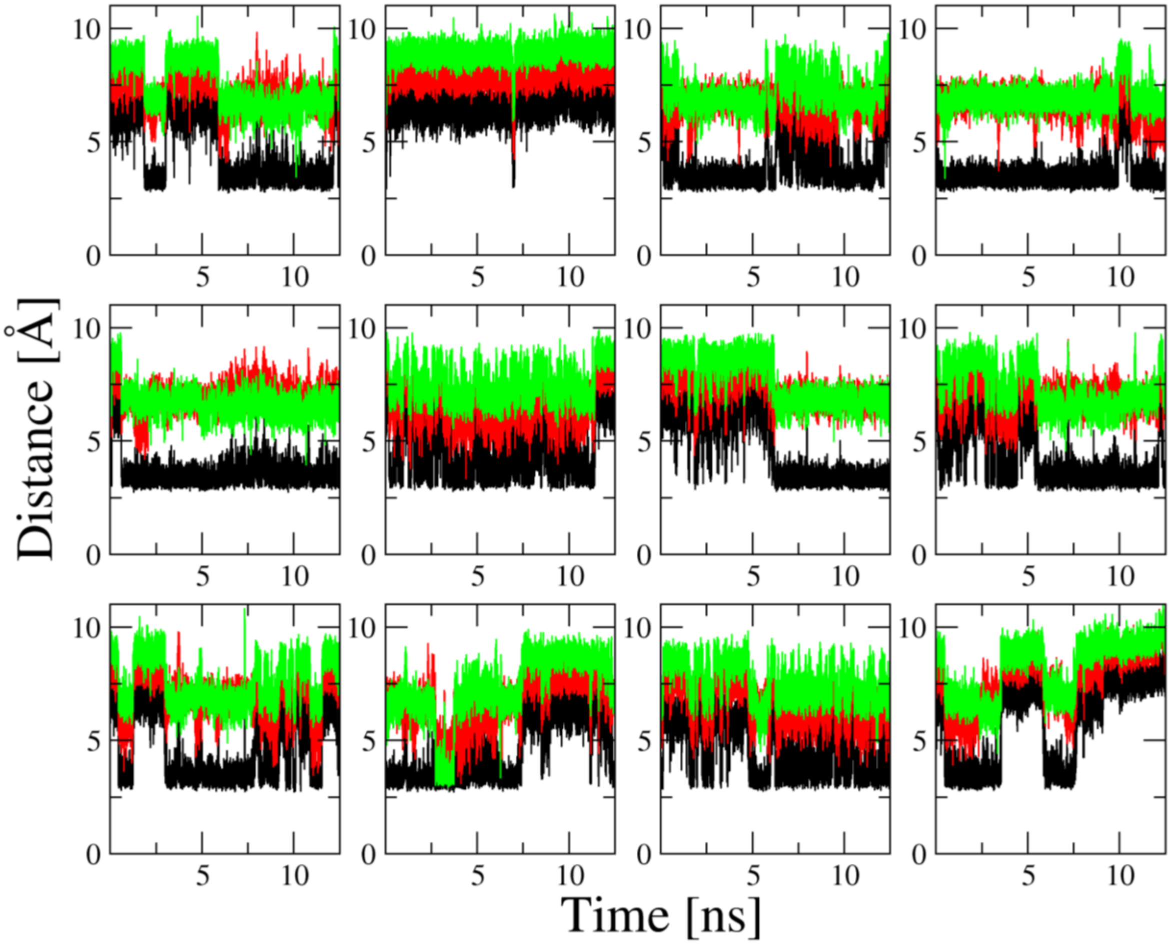

Figure S2. Ground-state MM/MD trajectories of the shortest CO-W124(indole) distances in **Re126W124W122Cu^{I}**. Black, green: equatorial CO ligands. Red: axial CO. The trajectories are in the same order as in Figure S1.

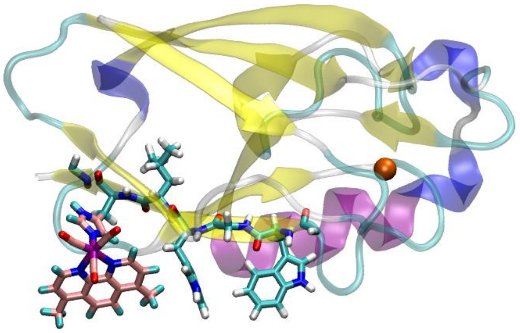

A: Crystal structure, "*in*"

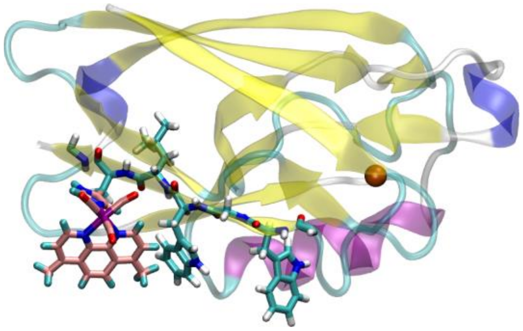

B: "*in*"; W124-W122 distorted

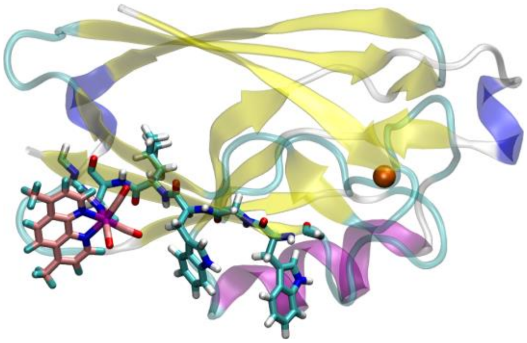

C: "*out*"

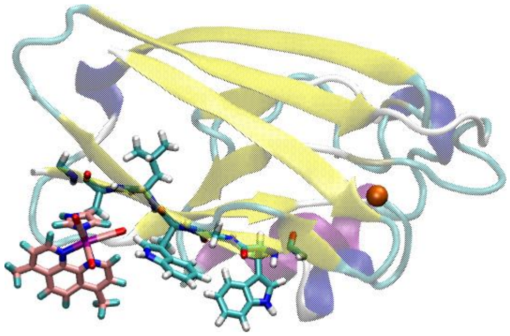

D: "*out*"; W124-W122 distorted

Figure S3. Selected ground-state **Re126W124W122Cu^{I}** structures. A: Crystal structure[1] is a prototypal reactive configuration. B-D show that the indole-indole distance and orientation vary in both conformations. The **Re**-H126-L125-W124-G123-W122 unit is shown as a stick representation.

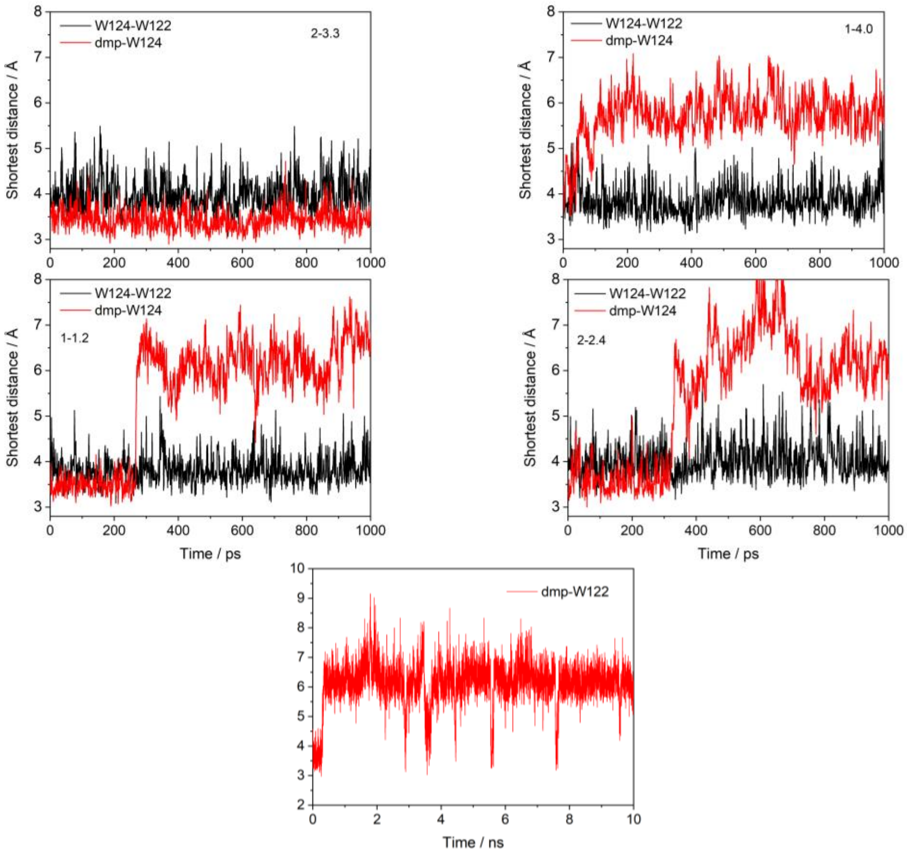

Figure S4. Top four panels: Typical CS1 MM/MD unreactive trajectories of **Re126W124W122Cu^{I}**. Black: shortest distance between W122 and W124 aromatic-ring atoms. Red: shortest distance between W124(indole) and dmp atoms. Top-left trajectory (2-3.3) stays the whole time in the "*in*" conformation (indole-indole mean distance 4.0 Å; indole-dmp mean distance = 3.4 Å). The other three trajectories show conversion to the "*out*" conformation.

Bottom: A 10-ns trajectory showing short-lived "*out*"→"*in*" conversions.

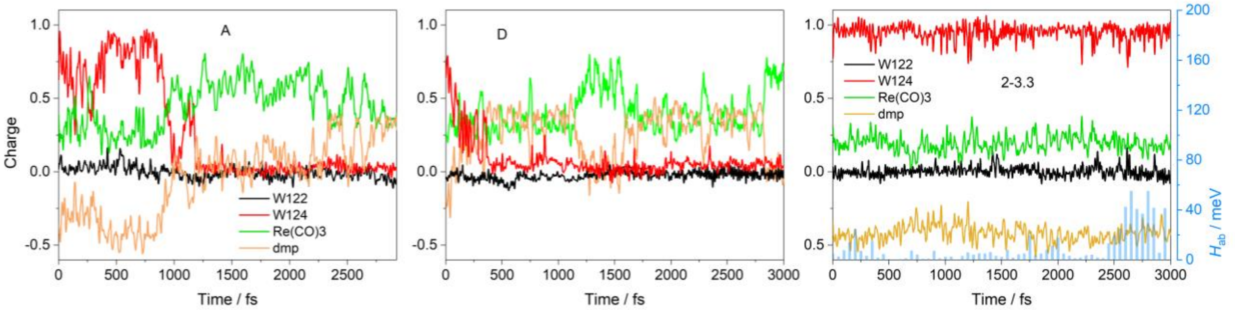

Figure S5. Left and middle: CS1 QM/MM/MD charge trajectories (A, D) showing reverse ET to *Re with a predominantly ππ*(dmp) electronic structure. Right: a typical unreactive "*in*"-CS1 QM/MM/MD charge trajectory (2-3.3).

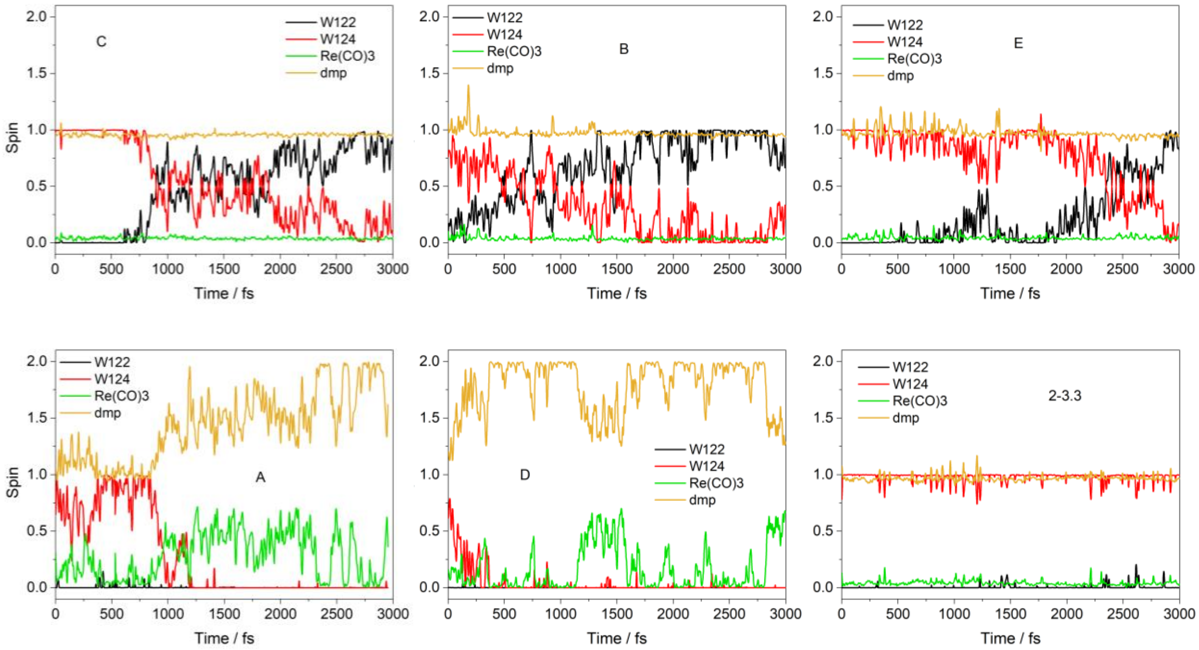

Figure S6. Evolution of spins at the two indoles (W124, W122), $Re(CO)_3$, and dmp along CS1 UKS QM/MM/MD trajectories. Top row: Reactive trajectories showing conversion to CS2. Bottom left and middle: Trajectories showing reverse ET to *Re of predominant $^3\pi\pi^*$ IL(dmp) character. Bottom right: Typical unreactive trajectory of a CS1 state in the "*in*" conformation (2-3.3). Letters specify the starting ground-state structures (Figure 2).

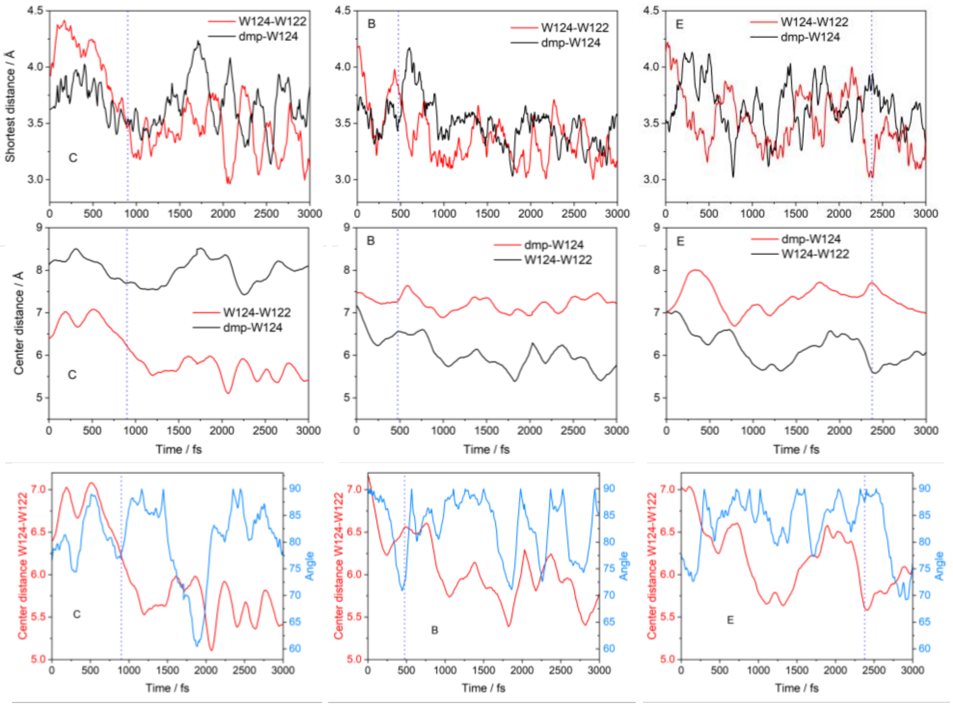

Figure S7. Distances and angles along reactive trajectories. Top: Shortest indole-indole (red) and W122-indole – dmp distances (black). From Figure 7. Middle: Center-center indole-indole (red) and W122-indole – dmp distances (b;ack)(from Figure 7). Bottom: Center-center distances (red) and angles between the two indoles.

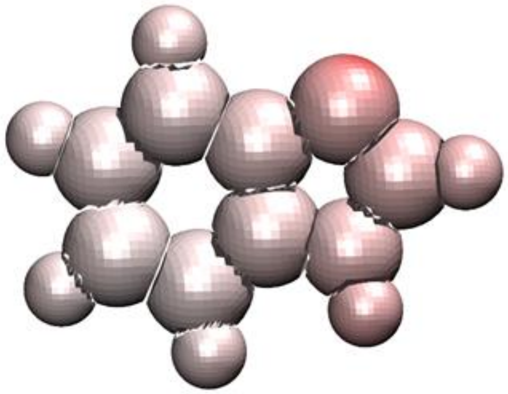

Figure S8. Example of an indole surface used in electrostatic potential calculations. Further details are provided in Section S1.5.

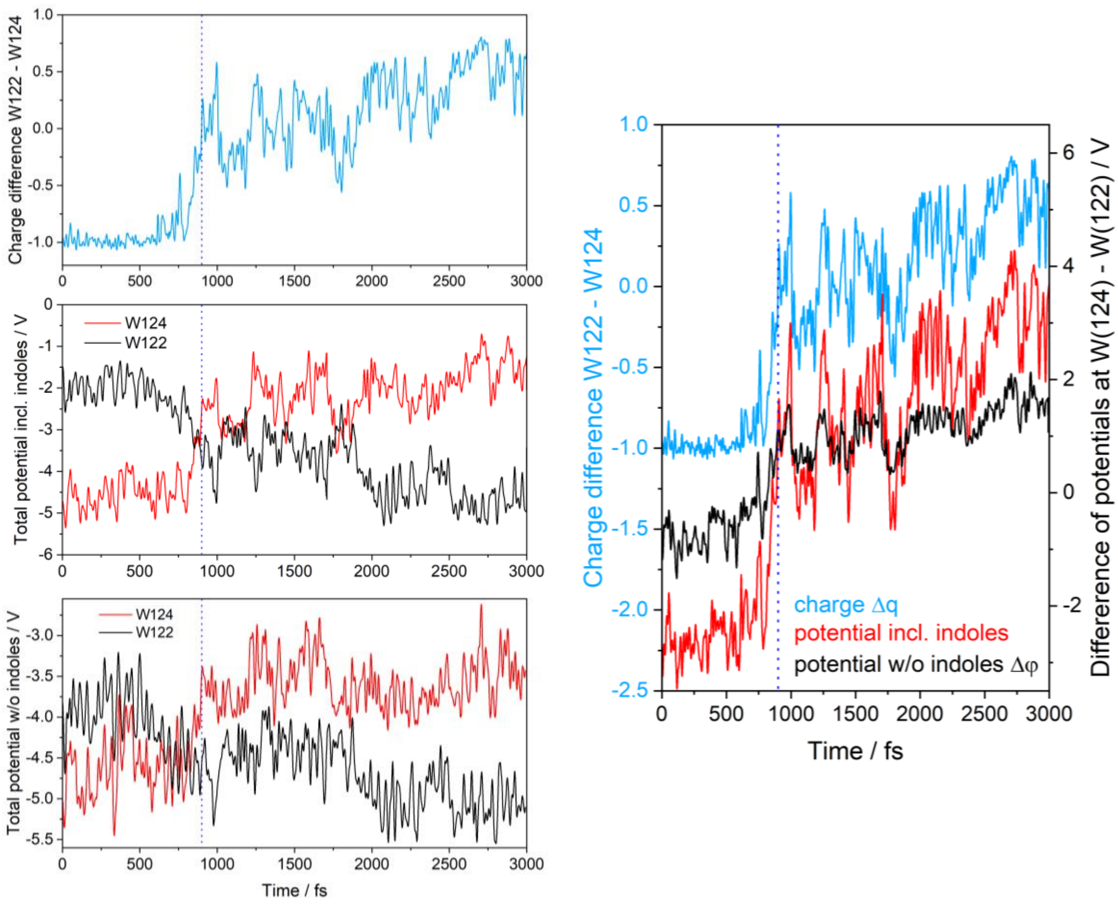

Figure S9. Temporal evolution of charge and potentials along trajectory C.
Left-top: Difference between W122 and W124 charges (Δq, blue). Left-middle: Electrostatic potentials at W124 and W122 generated by all atoms in the system including the other indole.
Left-bottom: Electrostatic potentials at W124 and W122 generated by all atoms in the system except the other indole (φ(124) and φ(122) as used in the text).
Right: Differences between charges (Δq, blue) and electrostatic potentials at the two indoles. Red: Total potentials generated by all atoms including the other indole. Black: Potentials generated by all atoms without the other indole (Δφ).
The dotted vertical line marks the time when the charges at W124 and W122 became equal for the first time.

Potential trajectories with and without indoles follow the same trends; and they both cross at the onset of the ET region. (To calculate potentials at W124 except the other indole, W122 was removed from the system, and vice versa. In this way, we excluded electrostatic effects of the shifting charge.)

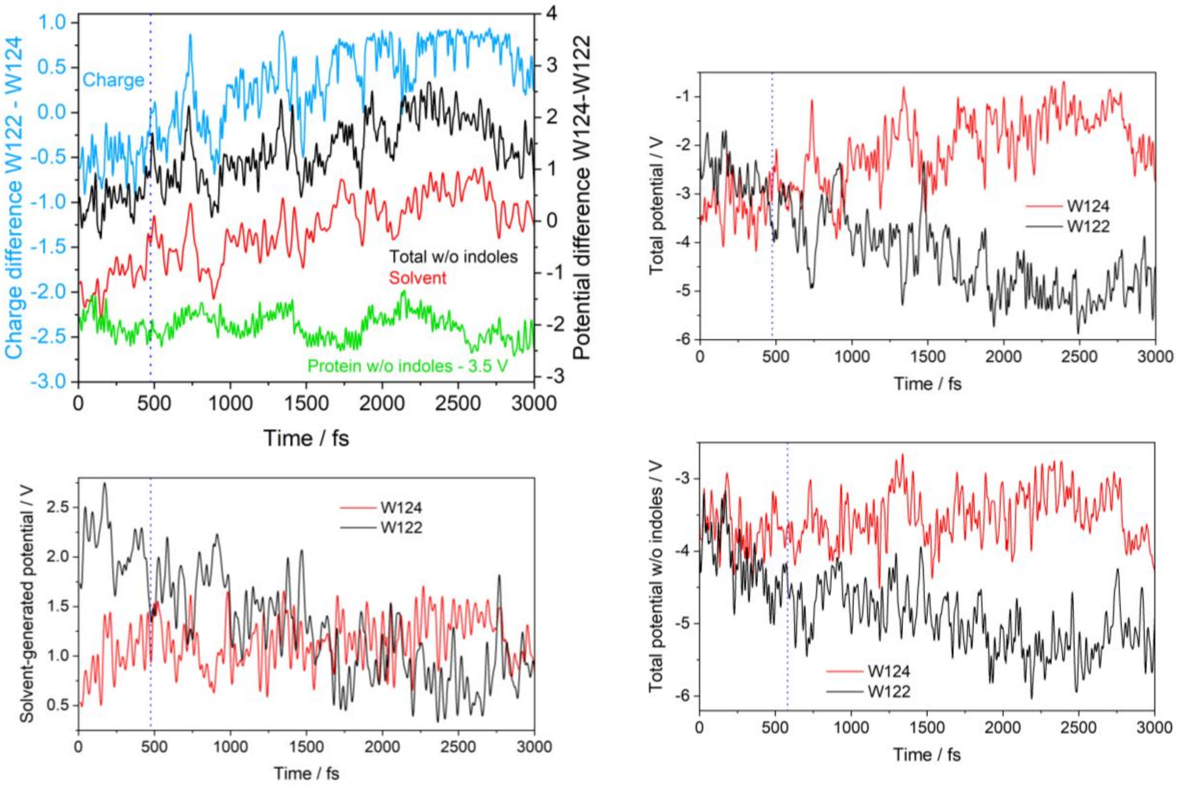

Figure S10. Temporal evolution of charges and electrostatic potentials along trajectory B. Left-top: Difference of electrostatic potentials at W124 and W122 generated by all atoms in the system except the other indole ($\Delta\varphi$, black), by the solvent ($\Delta\varphi$(solvent), red), and by the protein except the other indole ($\Delta\varphi$(prot), green, shifted by -3.5 V (B) for clarity). The charge difference between W122 and W124 (Δq) is shown in blue.

Left-bottom: Solvent-generated potentials at W124 and W122.

The dotted vertical line marks the time when the charges at W124 and W122 became equal for the first time.

Right: Electrostatic potentials at W124 and W122 generated by all atoms (top) and except the other indole (bottom).

Note that potential trajectories with and without the other indole follow similar trends, especially that they both cross at the onset of the ET region. (To calculate potentials at W124 without the other indole, W122 was removed from the system, and vice versa. This way, we have excluded electrostatic effects of the shifting charge.)

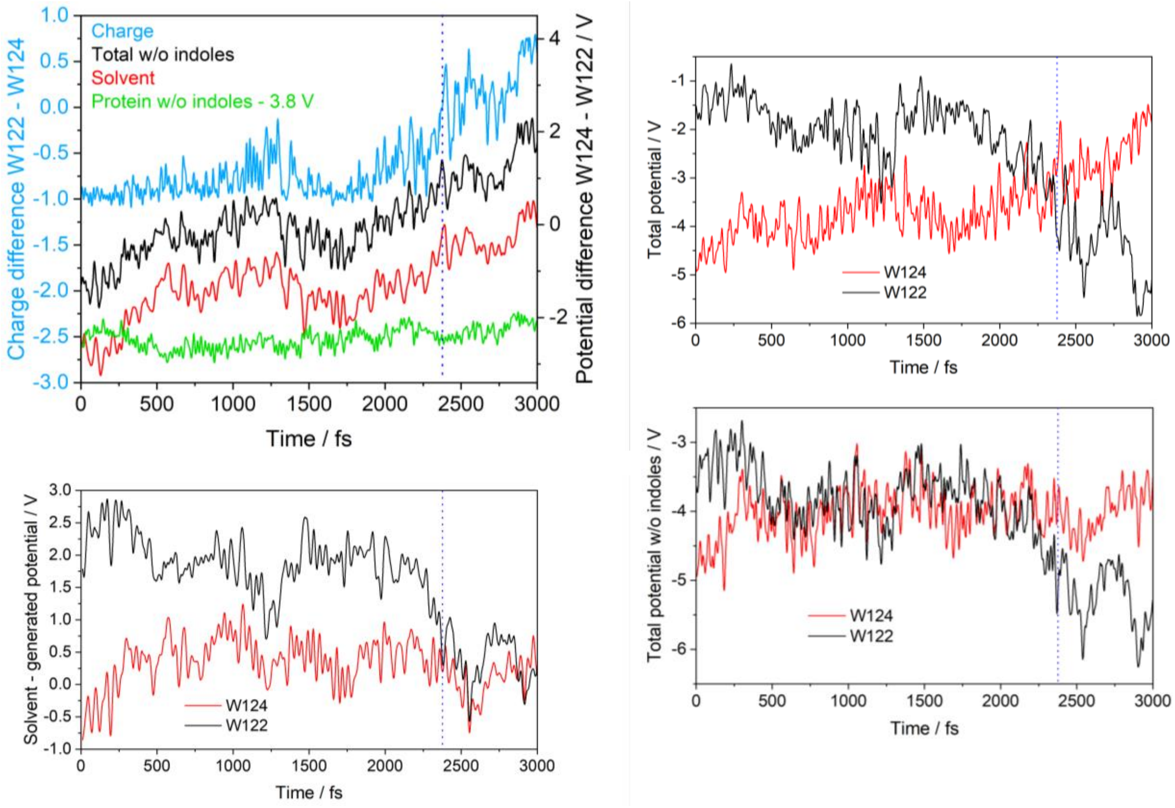

Figure S11. Temporal evolution of charges and electrostatic potentials along trajectory E.
Left-top: Difference of electrostatic potentials at W124 and W122 generated by all atoms in the system except the other indole (Δφ, black), by the solvent (Δφ(solvent), red), and by the protein except the other indole (Δφ(prot), green, shifted by -3.5 V (B) for clarity). The charge difference between W122 and W124 (Δq) is shown in blue.
Left-bottom: Solvent-generated potentials at W124 and W122.
The dotted vertical line marks the time when the charges at W124 and W122 became equal for the first time.

Right: Electrostatic potentials at W124 and W122 generated by all atoms (top) except the other indole (bottom).

Note that potential trajectories with and without the other indole follow similar trends; and they both cross at the onset of the ET region. (To calculate potentials at W124 without the other indole, W122 was removed from the system, and vice versa. This way, we have excluded electrostatic effects of the shifting charge.)

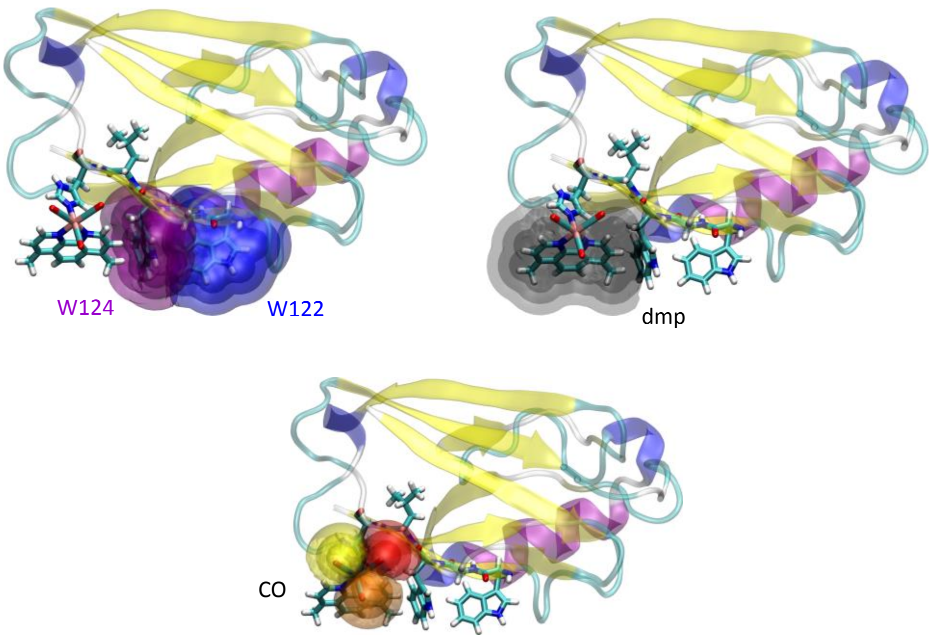

Figure S12. Non-overlapping proximal volume shells around individual fragments used to calculate water proximal radial distribution functions g(*r*) and coordination numbers.[2-4] Top-left: Shells around the two indoles, which compete for water among themselves but not with other fragments. Top-right: Shell around dmp that does not compete with any other fragment. Shells around the three CO ligands that compete for water among themselves but not with other fragments.

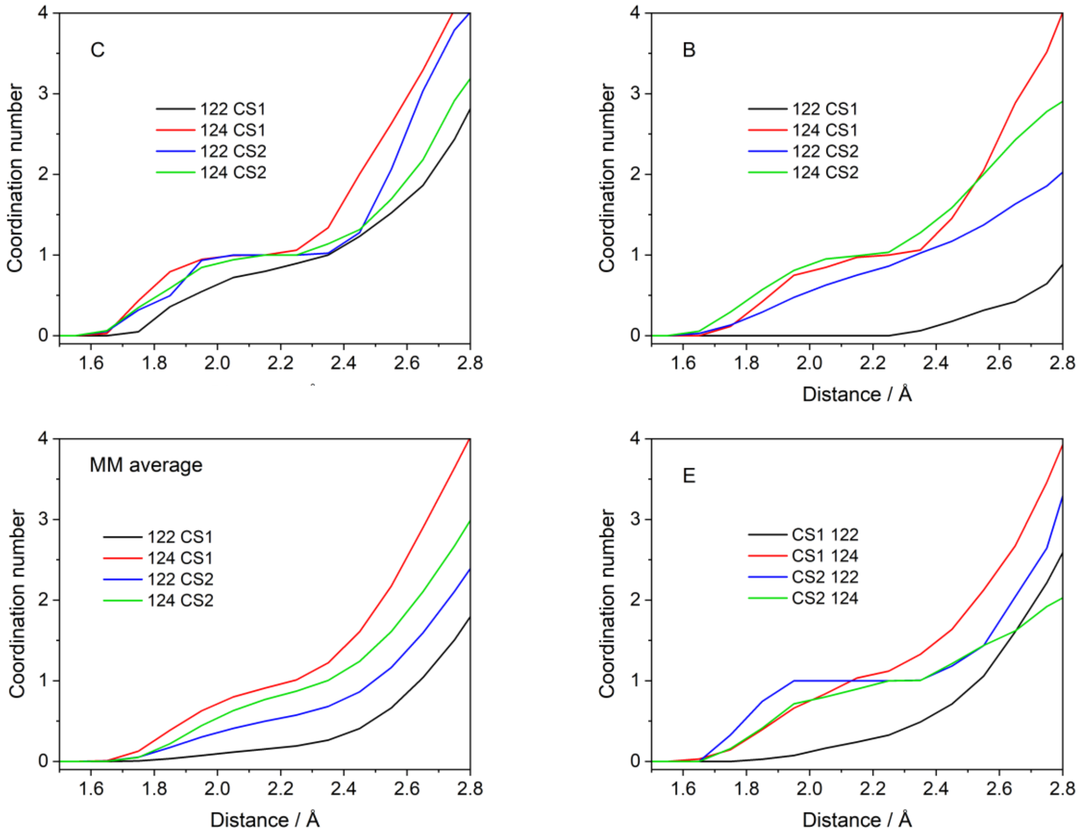

Figure S13. Water coordination numbers of W122 and W124 calculated from CS1 and CS2 parts of the reactive trajectories (clockwise from top-left) and averaged MM/MD trajectories of CS1 and CS2 (bottom-left). Each water molecule was assigned solely to its closest residue. The corresponding non-overlapping proximal volume shells are depicted in Figure S12.

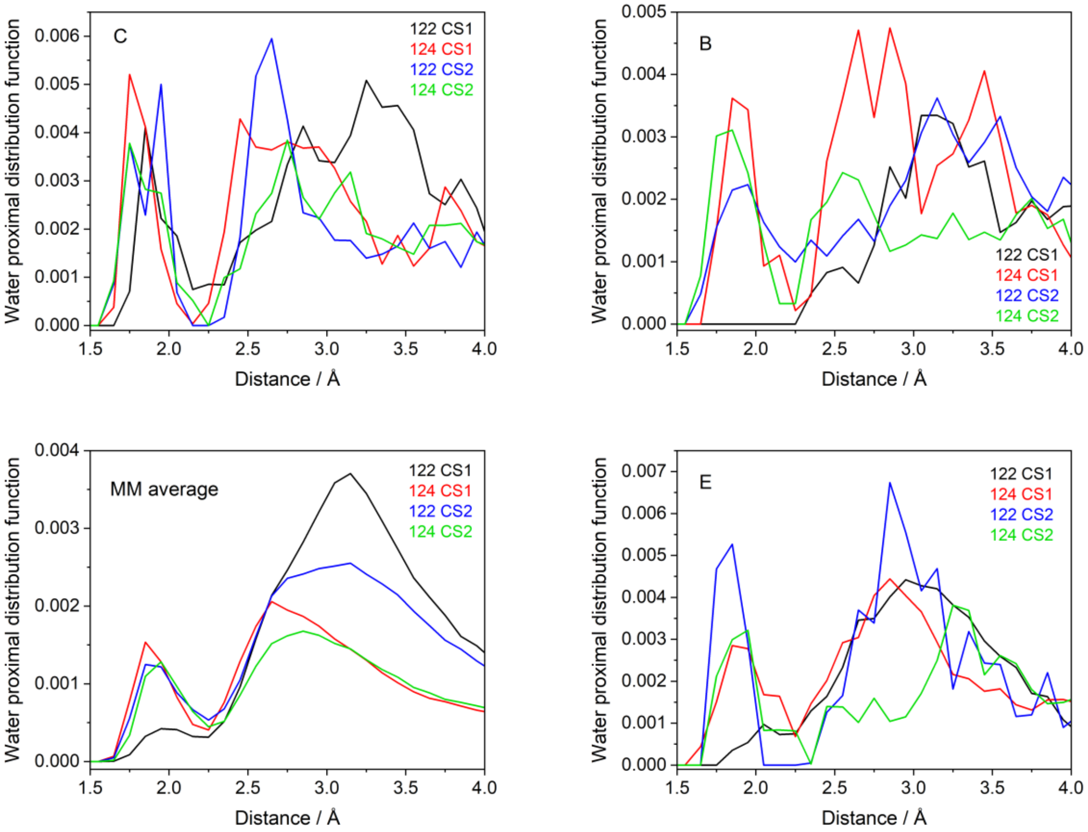

Figure S14. Water proximal distribution functions of W122 and W124 calculated from CS1 and CS2 parts of the trajectories (clockwise from top-left) and averaged MM/MD trajectories of CS1 and CS2 (bottom-left). Each water molecule was assigned solely to its closest residue. The corresponding non-overlapping proximal volume shells are depicted in Figure S12.

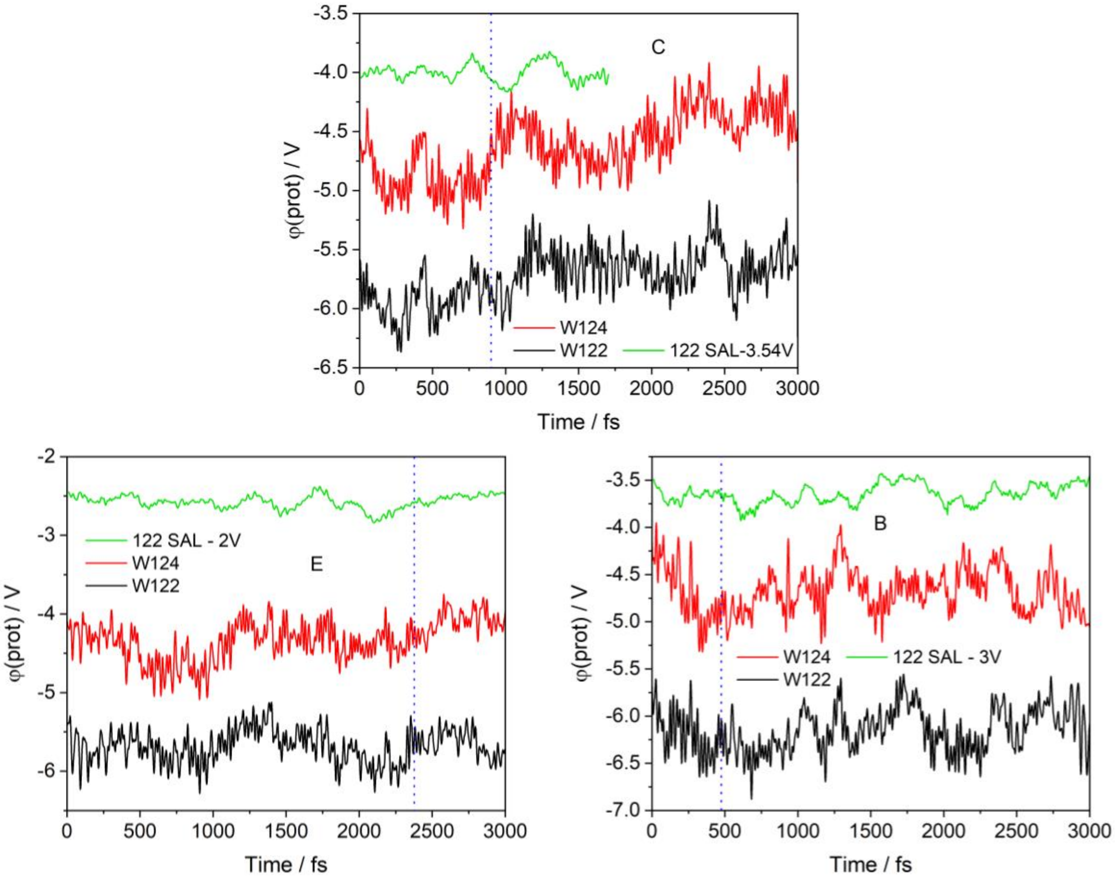

Figure S15. Electrostatic potential at W124 (red) and W122 (black) generated by the protein. Green: potential at W122 generated by the S118A119L120 segment.
("Protein" contains **Re**$^{-}$ but not the other indole.)

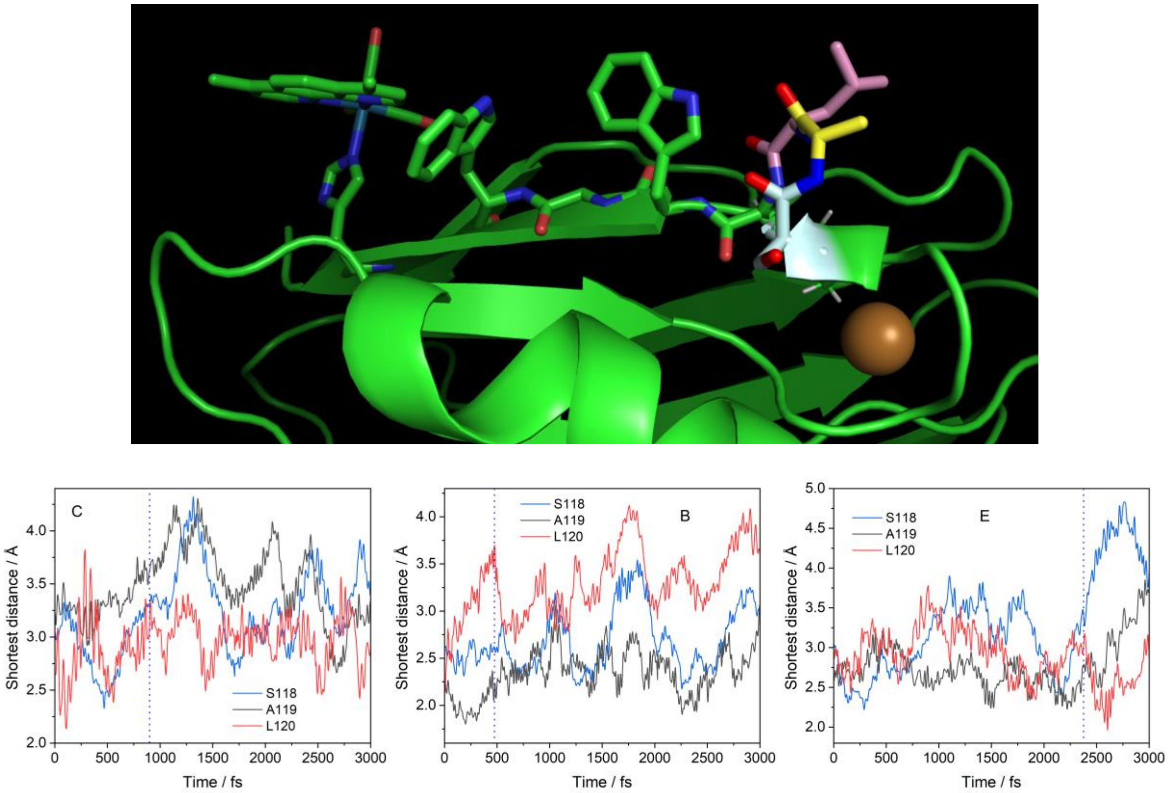

Figure S16. Top: Shielding of W122 by the SAL segment. S118 light-blue, A119 yellow, L120 pink. (See also Figure 8.)

Bottom: Shortest distances between W122 and individual residues of the SAL segment. ET starts in the region of increasing distances to all three residues (blue dotted line). Hydrogen atoms were included in distance calculations.

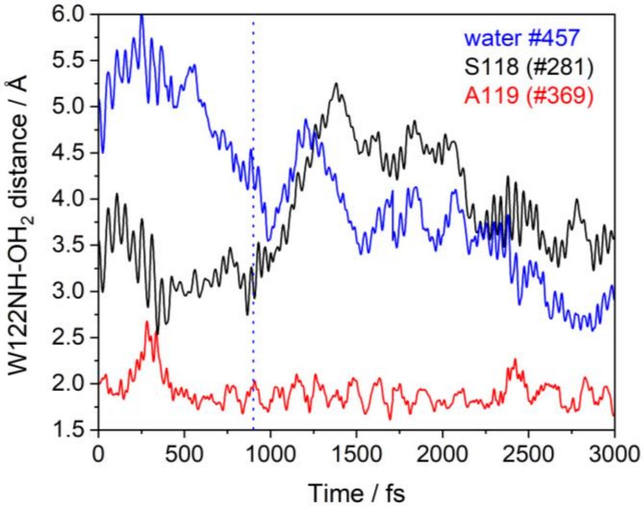

Figure S17. Evolution of the W122 indole-NH – OH_2 distance for the water molecule making a bridge to the A119 amide-O atom (red, water #369), to the first water molecule of the bridge to the S118 amide-O atom (black, water #281); and to a water molecule #457 (blue) that approaches in the direction from W124. Calculated along trajectory C.

The A119 amide-O···H_2O···HN-W122 bridge emerged ca. 400 fs before the ET onset and stayed stable through the ET and CS2 regions of the trajectory. The S118 amide-O···H_2O···H_2O···HN-W122 chain also formed ca. 400 fs before the ET onset. The φ(122-solv) decrease was reinforced by another H_2O molecule (#457, blue) that approached in the direction from W124. The S118 amide-O···H_2O···H_2O···HN-W122 chain opened up later in the ET region when water #281 moved away, together with #457, as SAL shifted away from W122. However, the combined electrostatic field of these two molecules kept the φ(122-solv) low. Both these water molecules started moving back toward W122 after ~1250 fs (#457) and ~1500 fs (#281), helping to drive the ET to completion and stabilizing CS2 at ≥2000 fs.

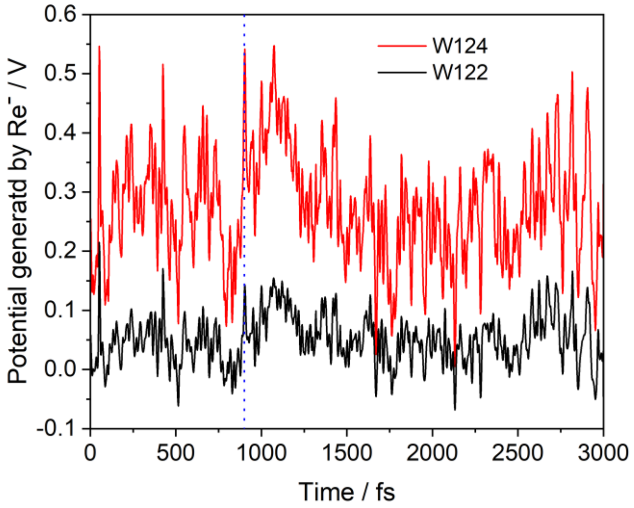

Figure S18. Electrostatic potential at W124 (red) and W122 (black) generated by **Re⁻** along trajectory C.

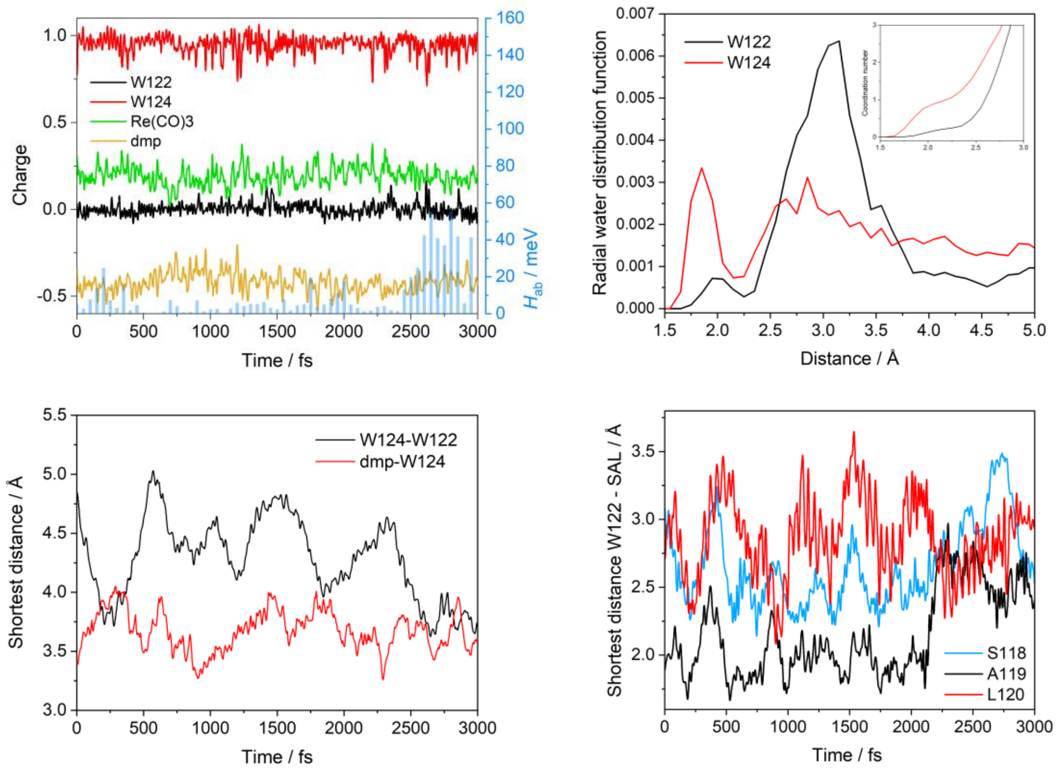

Figure S19. Characteristics of the unreactive trajectory 2-3.3, where **Re**$^{-}$ keeps the "*in*" orientation and the indoles are relatively close to each other.

Top - left: Charges at molecular fragments and CS1-CS2 electronic coupling

Bottom - left: Shortest indole-indole and W122-dmp distances (H atoms not considered)

Top - right: Proximal radial water distribution functions around W124 and W122 indoles.

Inset: Water coordination number up to 3 Å. Compare with Figure S14.

Bottom - right: Shortest distances between the W122 indole and SAL. Compare with Figure S16.

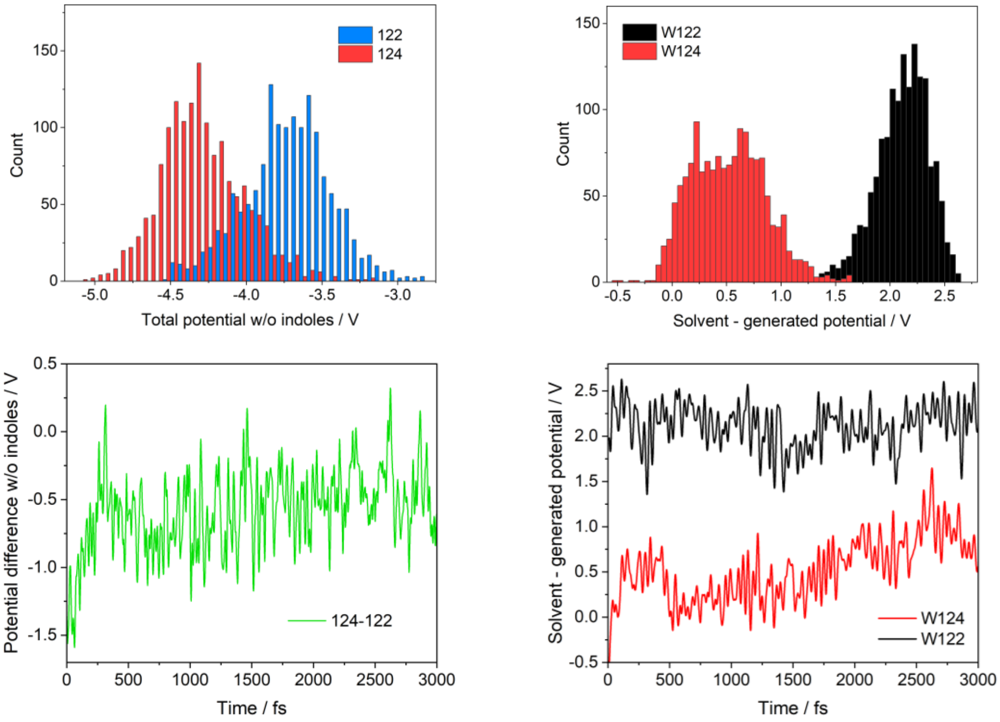

Figure S20. Electrostatic potentials calculated from the unreactive trajectory 2-3.3, where **Re⁻** keeps the "*in*" orientation and the indoles are relatively close to each other.

Left-top: Distributions of φ(124) and φ(122) over the 3 ps QM/MM/MD trajectory. The maxima are separated by ca. 0.8 V. φ(124) is more negative, stabilizing $W124^{\bullet+}$ (CS1).

Left-bottom: The difference between potentials at the two indoles, φ(124) - φ(122) along the QM/MM/MD trajectory stays <0.5, that is always below the 1.1 V level where the ET occurs.

Right-top: Distributions of φ(124-solv) and φ(122-solv) over the 3 ps QM/MM/MD trajectory. The maxima are separated by ca. 1.7 V. φ(124-solv) is more negative, stabilizing $W124^{\bullet+}$ (CS1).

Right-bottom: φ(124-solv) and φ(122-solv) along the 3 ps QM/MM/MD trajectory. φ(124-solv) << φ(122-solv) all the time, stabilizing $W124^{\bullet+}$ (CS1). The solvent-generated potentials never equalized in the course of the 3 ps simulation.

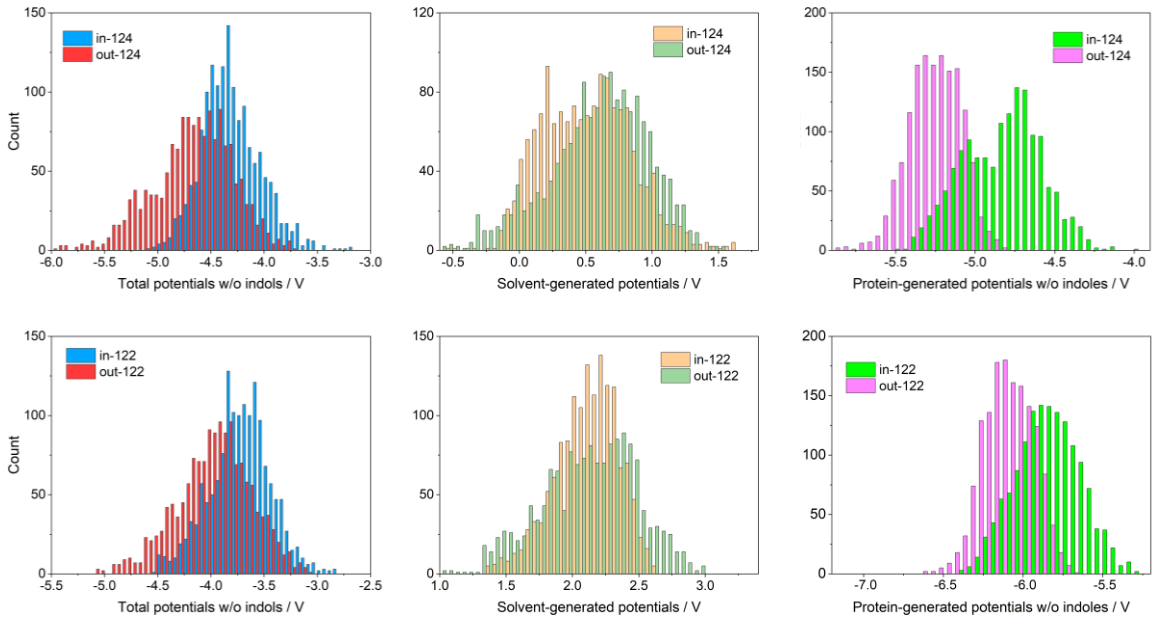

Figure S21. Distributions of electrostatic potentials at W124 (top) and W122 (bottom) indoles over "*in*" (2-3.3) and "*out*" (2-2.4) unreactive QM/MM/MD trajectories. The width of the potential range is the same in each column.

Left column: On going from "*in*" to "*out*" conformation, the distribution of total potentials at both indoles shifts lower but more at W124, resulting in a more stable CS1 relative to CS2.

Middle column: On going from "*in*" to "*out*" conformation, the distribution of solvent-generated potentials at W124 shifts slightly higher than at W122. Hence, solvation stabilizes *in*-CS1 slightly more than *out*-CS1.

Right column: On going from "*in*" to "*out*" conformation, the distribution of protein-generated potentials at both indoles shifts lower but much more at W124, resulting in strong CS1 stabilization relative to CS2. Protein-generated electrostatic potential is responsible for the overall out-CS1 stabilization indicated by the left column.

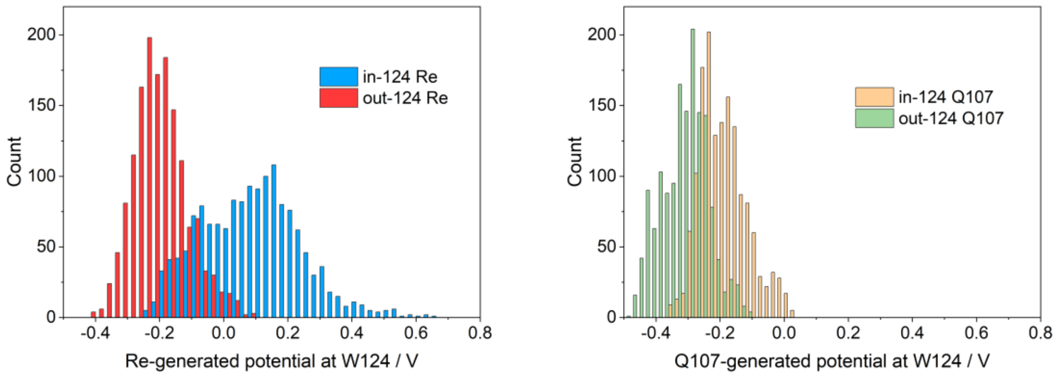

Figure S22. Distributions of electrostatic potentials at W124 (left) and W122 (right) indoles over "*in*" (2-3.3) and "*out*" (2-2.4) unreactive QM/MM/MD trajectories generated by **Re**⁻ and Q107. The potential range is the same in each panel.

Electrostatic potentials generated at W124 by **Re**⁻ and, to a lesser extent, Q107 are lower in the "*out*" than the "*in*" form. **Re**⁻ and, to a lesser extent, Q107 are the main contributors to the *out*-CS1 stabilization relative to "*in*" by the protein-generated potentials shown in Figure S22.

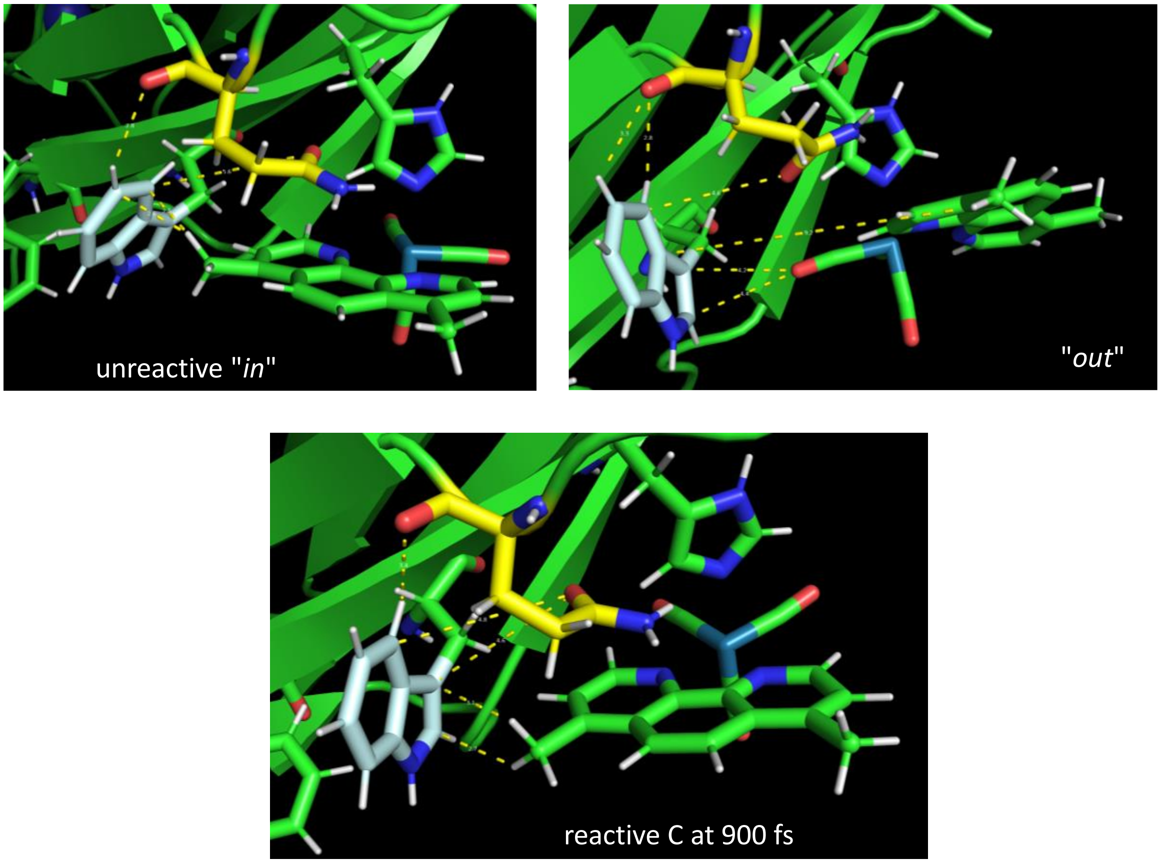

Figure S23. Relative orientations of the W124 indole (light blue), **Re**$^{-}$ (green) and Q107 (yellow).
Top-left: unreactive "*in*" QM/MM/MD trajectory 2-3.3 at 2010 fs.
Top-right: unreactive "out" QM/MM/MD trajectory 2-2.4 at 2010 fs.
Bottom: reactive QM/MM/MD trajectory C at 900 fs (ET start time).

The Q107 peptide O-atom is relatively close to W124 in all cases. The amide side chain is tilted toward the dmp$^{\bullet-}$ in the reactive (C) as well as unreactive "*in*" cases. In the "*out*" form, it is tilted toward W122. The corresponding O-W122 distance (4.2 Å to nearest C-atom) is shorter than in unreactive "*in*" and reactive cases.
Positively charged H-atoms of one of the dmp$^{\bullet-}$ CH_3 groups are very close to W122 in C and unreactive "*in*" (2.6-2.7 Å) and much farther in "*out*" (9.2 Å). Moreover, one of the C≡O ligands in the "*out*" form points toward W124. The closest distance between the negatively charged (ca. -0.17 e) O atom and W124 is 4.2 Å. These structural differences result in a much smaller electrostatic potential at W124 generated by **Re**$^{-}$ in the "*out*" than "*in*" conformer.

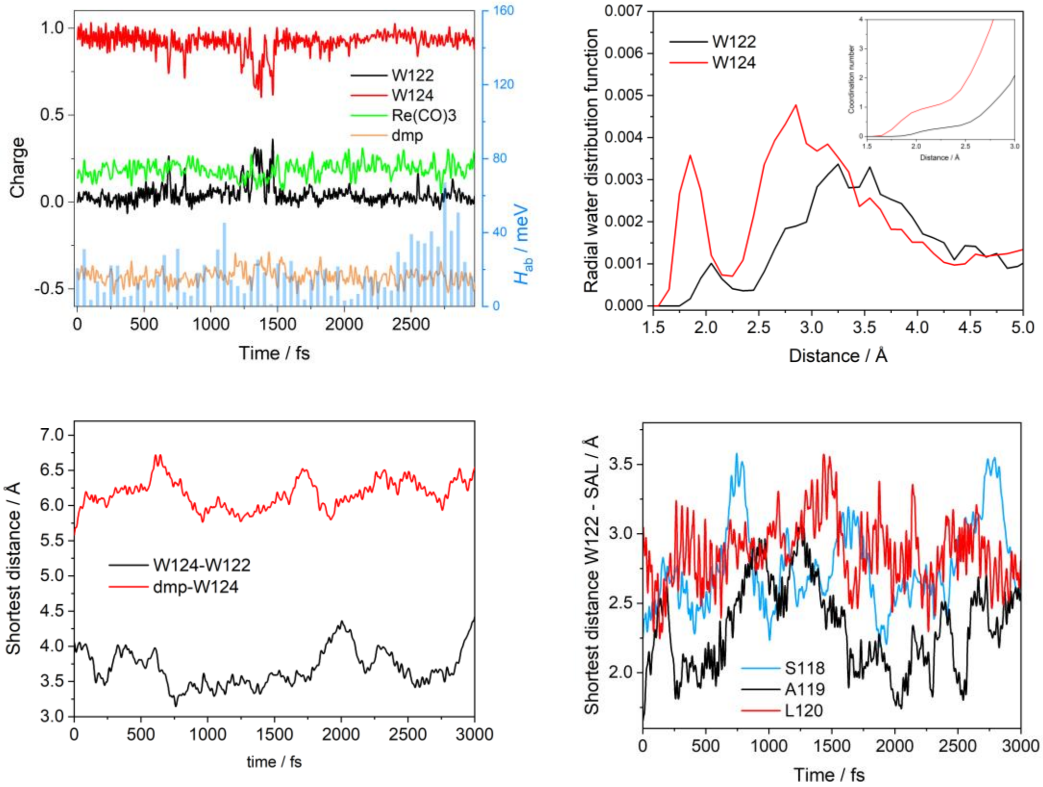

Figure S24. Characteristics of the unreactive trajectory 2-2.4, where **Re**$^{-}$ assumes the "*out*" orientation and the indoles are relatively close to each other.

Top - left: Charges at molecular fragments and CS1-CS2 electronic coupling

Bottom - left: Shortest indole-indole and W122-dmp distances (H atoms not considered)

Top - right: Proximal radial water distribution functions around W124 and W122 indoles.

Inset: Water coordination numbers up to 3 Å. Compare with Figure S14.

Bottom - right: Shortest distances between the W122 indole and SAL. Compare with Figure S16.

Generally, unreactive "*out*" and "*in*" trajectories are very similar. W124 is more solvated in the second solvation sphere, W122 less in the case of the "*out*" form. Compare with Figure S19.

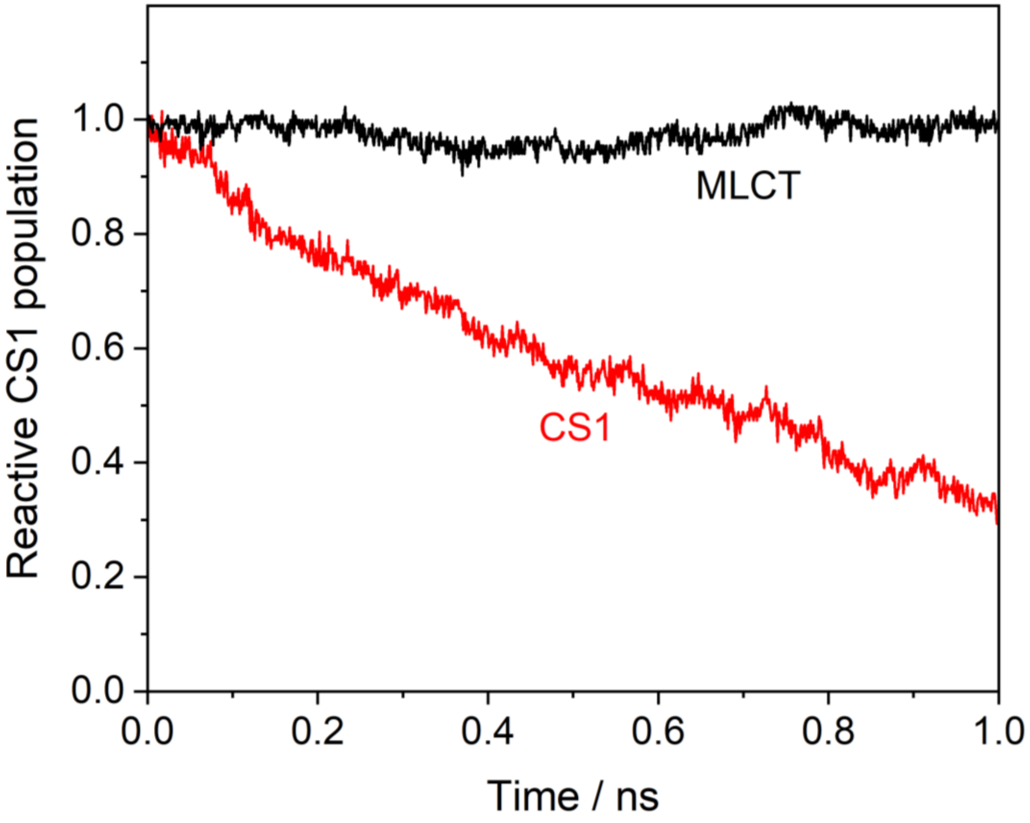

Figure S25. Temporal evolution of "*in*"-MLCT and "*in*"-CS1 populations from all calculated MM/MD trajectories. The "*in*" geometry was defined as dmp–W124 < 5 Å.

S1. Computational details

S1.1. General procedure.

The simulation protocol is summarized in the main text, Figure 1. In order to realistically simulate the evolution of CS1 and CS2 states by QM/MM, we prepared the initial thermalized (300 K) geometries by mimicking the experimentally established mechanism (Figure 1). We first performed 12.5 ns MM/MD *NpT* simulations with GS parameters to generate a set of independent geometries of the protein native state, sampling different sidechain conformations and solvent distributions. Next, starting from hundreds of randomly selected geometries, we performed 1 ns long MM/MD simulations with MLCT FF parameters, followed by 1 ns long MM/MD simulations with CS1 FF parameters. Simulation lengths were restricted to 1 ns since long propagation of excited states (significantly longer than their lifetimes) by classical force-fields may lead to population of unrealistic (over-relaxed) geometries that would bias both the ensemble averages and available reactive pathways. The final geometries and velocities of MM simulations served as inputs for excited-state UKS QM/MM/MD runs. Structural parameters were monitored in all simulations (see below). UKS simulations were used to study the ET reactivity by following the charge and spin transfer between **Re**, W124, and W122. The same procedure was applied to investigate the CS2 state, whose MM/MD simulations started from CS1 MM/MD structures (Figure 1, main text).

S1.2. Classical MM/MD simulations

We performed classical MM/MD simulations using AMBER 14 software and parameters[5] for the MM part of the system. In order to properly parametrize the QM-region, we employed a unique set of parameters that we have previously developed[4] for $[Re(imidazole)(CO)_3(dmp)]^+$. Atomic charges for CS1 and CS2 triplet states of the QM part were obtained as described in Section S1.8. The values are summarized in Table S1. For the rest of the protein, the ff14SB modifications of parm10 parameters[6, 7] were employed. The vicinity of the Cu^I atom was not investigated in this work. To keep a realistic geometry, we restrained the Cu-ligand distances using data from Table 1 of ref.[8] The SPC/E model was used for explicit water surroundings[9] and two Na^+ cations were added to neutralize the system. Since the Terachem-Amber QM/MM does

not support periodic boundary conditions (PBC), we employed harmonic potential on all water molecules to keep them in the spherical water "cap" (6683 water molecules in total) around the protein, and the whole solvated system was simulated as a large cluster. Electrostatics was accounted for by a very large Coulomb cut-off. MM/MD simulations of the total length of 60 ns were performed in an *NpT* ensemble at an ambient temperature and pressure (300 K, 1 bar) controlled by a Berendsen thermostat and barostat, employing periodic boundary conditions and a 1 fs time-step.[10] Relevant MM/MD trajectories are exhibited in Figures 2, S1, S2 (ground state) and S4 (^{3}CS1 excited state).

S1.3. QM/MM/MD simulations

The **Re126W124W122Cu^I** system was divided into QM (quantum) and MM (classical) parts as shown in Figure 1. The QM region was defined as $Re(CO)_3$(dmp)(H126)L125W124G123W122. It was terminated by linking-H-atoms that were attached to corresponding C_α atoms of the protein backbone. The rest of the system formed the MM region. The protein construct was solvated in a cubic box with 10360 SPC/E[9] water molecules, resulting in a minimum 12 Å water shell. Two Na^+ cations were added to compensate the charge of the protein.[11] Initial structures were based on the pdb 6MJS.

Molecular dynamics (MD) simulations of the **Re126W124W122Cu^I** lowest triplet state was performed at the QM/MM level in Terachem 1.9[12, 13] – Amber 14[5] framework using electronic embedding. Description of the MM part remained unchanged and QM-part calculations utilized LANL2DZ quasi-relativistic effective core pseudopotentials and the corresponding optimized set of basis functions for Re[14] and 6-31g(d) polarized double - ζ basis sets for remaining atoms.[15] DFT calculations employed the hybrid functional PBE0[16, 17] together with an empirical dispersion correction (D3).[18] CS1 and CS2 triplet states were calculated by the unrestricted KS procedure (UKS). Several testing calculations were performed with the long-range-corrected functional CAM-B3LYP,[19] see section S1.4.

Excited-state QM/MM/MD simulations were performed with a 1 fs time step using the SHAKE algorithm.[20] Production runs were performed at 300 K employing the Berendsen thermostat.[10] The CM-motion was removed every 1 ps. Simulation were run for 3 ps.

S.1.4. CAM-B3LYP test calculations

In order to verify that the structural conditions for CS1 and CS2 states established with the PBE0 functional are realistic, short QM/MM/MD simulations using the long-range-corrected functional CAM-B3LYP[19] were performed, starting from regions of reactive CS1 trajectories where PBE0 calculated either CS1 or CS2 electronic structure. In accord, CAM-B3LYP provided the same electron density distributions as PBE0, which remained stable in the course of following CAM-B3LYP QM/MM/MD dynamics. This is demonstrated in Figure S26 by CAM-B3LYP charge trajectories starting from CS1 and CS2 areas of the reactive trajectory C.

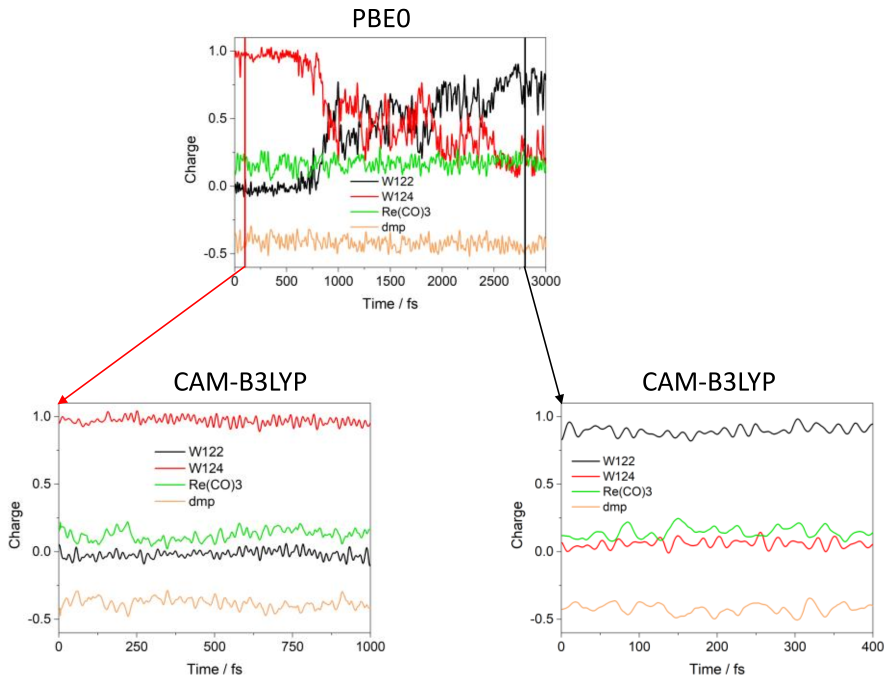

Figure S26. Top: Evolution of charges at the two indoles, $Re(CO)_3$ and dmp along the reactive CS1 UKS QM/MM/MD trajectory C calculated using the PBE0 functional (from Figure 4). Bottom: evolution of charges along UKS QM/MM/MD trajectories calculated with CAM-B3LYP. The starting structures were taken from the trajectory C at the times marked by red (100 fs, CS1) and black (2800 fs, CS2) vertical lines.

S1.5. Electronic coupling

Calculations of electronic coupling H_{ab} between W122 and W124 fragments were carried out in the Q-Chem 6.0 program package using methods of constrained DFT (CDFT-CI and absolutely localized molecular orbitals (ALMO/DFT)[21, 22]. DFT (CDFT-CI) calculations at the PBE0-D3(BJ)/6-31G(d) level were performed on systems where the QM part consisted of methylindoles from

W122 and W124 amino acids. Effects of the rest of protein and solvent surroundings were included by adding electrostatic interactions with point charges from the MM part into the QM Hamiltonian. The regarded states were determined by fixing both the +1 e charge and the ½ spin on either W122 (CS2) or W124 (CS1). Calculations were performed at selected snapshots of QM/MM/MD trajectories. Results obtained by CDFT-CI and ALMO/DFT are compared and the effect of the functional are shown in Figure S27.

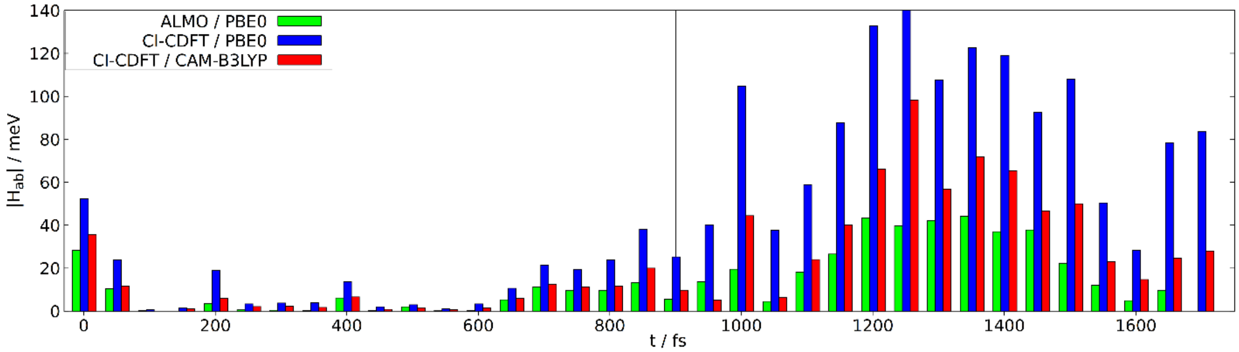

Figure S27. CS1-CS2 electronic coupling along the first half of the reactive trajectory C calculated by ALMO/PBEO (green), CI-CDFT/PBE0 (blue), and CI-CDFT/CAM-B3LYP (red). Results of all three types of H_{ab} calculations are qualitatively compatible with the conclusions in the main text.

S1.6. Electrostatic potentials

Electrostatic potentials *φ(r)* were determined based on the atomic charges q_i from the UKS/MM dynamics and calculated at a given point as $\varphi_A(\boldsymbol{r}) = \sum_i \frac{q_i}{|\boldsymbol{r}_i - \boldsymbol{r}|}$, where the sum runs over contributing set of atoms *A* (can be solvent, protein, etc.). If such atoms belonged to the QM part, their actual Mulliken charges from UKS were employed, otherwise charges from the MM force field were used. For the investigated fragment (active indole or **Re**), potentials were evaluated on a surface obtained as an envelope of scaled van der Waals spheres around its atoms as shown in Figure S8. The scaling factor 0.5 was used. Points for potential calculations were generated based on Fibonacci lattices for the spheres. Finally, average potentials were determined as weighed averages of the potentials over the surface with weights corresponding to areas assigned to them based on the Delaunay triangulation. Atoms of the examined fragment never contributed to the potential. Calculations were performed using our Fortran code.

S1.7. Proximal volume *V*(*r*), coordination number *N*(*r*), and distribution function *g*(*r*)

The proximal radial distribution function, pRDF *g*(*r*) provides a quantitative and easy way to describe and interpret a solution structure (*e.g.* hydration) near non-spherical molecules.[2, 4] The calculation is analogous to that of a radial distribution function and follows from $g(r) = \frac{\rho(r)}{\rho_{\mathrm{bulk}}}$. The knowledge of the proximal coordination number *N(r)* of solvent molecules and proximal volume *V(r)* (see Figure S12) is needed to determine the local density of the solvent $\rho(r) = \frac{\Delta N(r)}{\Delta V(r)}$, and thus its deviation from the bulk density ρ_{bulk}.

The proximal distance is the closest distance measured between the water molecule and the solute (or a solute functional group). In the proximal sense, the hydration layer of a thickness *r* faithfully follows the shape ('molecular surface') of the molecule. The proximity criteria can be used for the entire protein molecule or for selected functional groups (e.g., amino acid residues, sidechains, …). This makes pRDF particularly convenient for proteins, flexible polymers, or complex molecules that consist of numerous functional groups.[2-4]

In the current study, we focused on electron transfer between CS1 and CS2 states, which are determined by the electronic state of the two indoles. Therefore, hydration of W122 and W124 was monitored over time windows covering the CS1 and CS2 periods of reactive trajectories C, B, E or MM/MD CS1 and CS2 trajectories and quantified by *g(r)* and *N(r)*.

The proximal coordination number quantifies the hydration of a residue. Apart from monitoring its fluctuations for stable CS1 and CS2 states, it provides insight into changes in indole hydration during the transition from CS1 to CS2 state or vice versa. The 'nonexclusive' approach was used, *i.e.,* a single water molecule may contribute to more groups, but in general at different proximal distances. In the exclusive approach, which was adopted in this work, a single water molecule contributes only to the closest of competing groups (see Figure S12).

Analyses were performed using our in-house Python implementation on system configurations, which were saved every 1 fs, yielding approximately 3000 samples for analysis over a 3 ps simulation.

S.1.8. Atomic charges for the CS1 and CS2 states

MM parameters for describing MLCT, CS1, and CS2 states were prepared from ground-state parameters[4] by changing the charge distribution on the $Re(CO)_3$(dmp)(H126)L125W124G123W122 active part of the system (that is the QM part of QM/MM simulations). Derivation of the corresponding MLCT charge distribution is described in our previous paper.[4] CS1 and CS2 charges for the active part were determined by averaging CM5 charges (Truhlar-CM5) from single-point UKS/MM calculations at the PBE0-D3(BJ)/6-31+G(d) level for tens of structures of the entire system in a similar way. The system division to QM and MM parts was the same as in the production dynamics. Nevertheless, structure selection is crucial in this step since the surroundings significantly polarize the electron density distribution on the active part. To get plausible charge estimates for the CS1 and CS2 states, we utilized longer parts of TDDFT/MM/MD trajectories from our previous study[4] corresponding to the required electronic state. Frame selection started at least 200 fs after the crossing with the lowest ^{3}MLCT state in order to allow system relaxation in the CS1 or CS2 state. In this way, 152 structures (A-1: 71, B-1: 35, B-2: 35, and D-1: 11; trajectories labels from ref.[4]) for CS1 and 70 structures (A-2: 50, B-3: 6, F-1: 6, F-2: 8) for CS2 were collected. Final atomic charges for MM simulations (Table S1) were obtained by averaging calculated CM5 charges and smearing the exceeding charge over the active part. The latter ensues from substitution of MM frontier atoms by hydrogen link atoms in the QM/MM calculations and our effort to keep the total charge of the protein +2 (including **Re**). The total charges on active cofactors are 0.029 e (whole W122), 0.969 e (whole W124), and -0.080 e (**Re**$^-$) for CS1 and 0.6814 e (whole W122), 0.275 e (whole W124), and -0.009 e (**Re**$^-$) for CS2.

The use of UKS-calculated charges for parametrization is justified by the fact that classical MM simulations are followed by the UKS/MM dynamics and in this way the change of the system description from classical to QM should be relatively smooth. To verify the eligibility of parameters, we ran testing MM dynamics for CS1 and recalculated the CM5 charges as described above. The determined average values (W122: -0.024, W124: 0.897, **Re**$^-$: 0.096) remained similar to the used atomic charges. Distributions of cofactor charges along the trajectory CS1 are shown in Figure S28. In the case of CS2, the positive charge is more delocalized between W122 and

W124. Hence, we tested the influence of MM parametrization on CM5 charges when the CS2 charge distribution was obtained by swapping the atomic charges between W122 and W124 keeping CS1 MM parametrization, which led to more localized charges in MM simulations. However, the impact on the CM5 charges was not significant. Ultimately, the validity of used charges is confirmed by their matching with charges determined in the production QM/MM simulations.

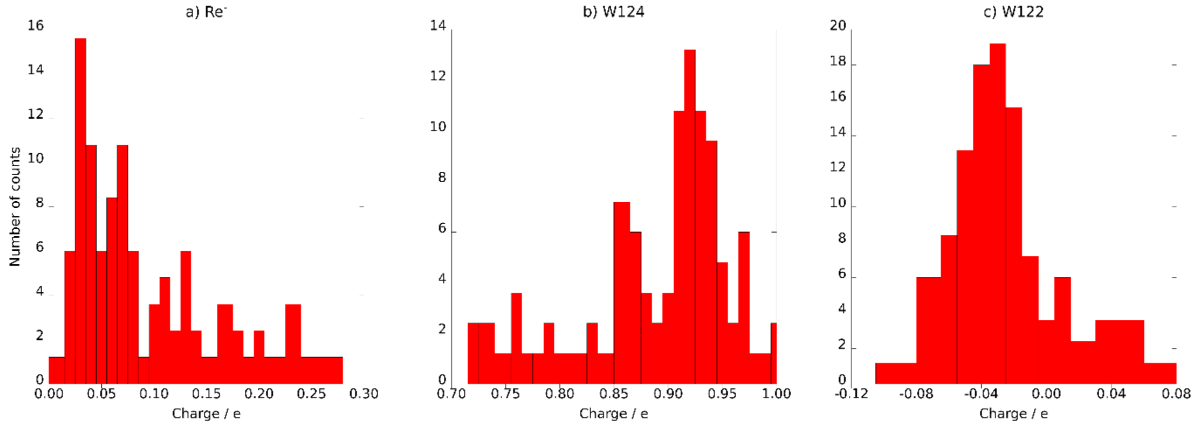

Figure S28. Distribution of total CM5 charges at the cofactors along 20 ns testing trajectory (89 structures analyzed) determined at PBE0-D3(BJ)/6-31+G(d) level with Stuttgart-Dresden pseudopotentials on the Re atom for a) **Re⁻**, b) W124, and c) W122.

Table S1. Atomic charges (in e) of the active part in MM simulations of GS, MLCT, CS1, and CS2 states.

Atom	Residue	Res. Number	GS	MLCT	CS1	CS2
N	MET	121	-0.4157	-0.4157	-0.4157	-0.4157
H	MET	121	0.2719	0.2719	0.2719	0.2719
CA	MET	121	-0.0237	-0.0237	-0.2146	-0.2135
HA	MET	121	0.0880	0.0880	0.1319	0.1350
CB	MET	121	0.0342	0.0342	0.0342	0.0342
HB2	MET	121	0.0241	0.0241	0.0241	0.0241
HB3	MET	121	0.0241	0.0241	0.0241	0.0241
CG	MET	121	0.0018	0.0018	0.0018	0.0018
HG2	MET	121	0.0440	0.0440	0.0440	0.0440
HG3	MET	121	0.0440	0.0440	0.0440	0.0440
SD	MET	121	-0.2737	-0.2737	-0.2737	-0.2737
CE	MET	121	-0.0536	-0.0536	-0.0536	-0.0536
HE1	MET	121	0.0684	0.0684	0.0684	0.0684
HE2	MET	121	0.0684	0.0684	0.0684	0.0684
HE3	MET	121	0.0684	0.0684	0.0684	0.0684
C	MET	121	0.5973	0.2608	0.2558	0.2626

O	MET	121	-0.5679	-0.4050	-0.3731	-0.3687
N	TRP	122	-0.4157	-0.4478	-0.4386	-0.4384
H	TRP	122	0.2719	0.3288	0.3370	0.3395
CA	TRP	122	-0.0275	0.0342	0.0352	0.0416
HA	TRP	122	0.1123	0.1249	0.1354	0.1413
CB	TRP	122	-0.0050	-0.1411	-0.1451	-0.1348
HB2	TRP	122	0.0339	0.1001	0.1100	0.1218
HB3	TRP	122	0.0339	0.1018	0.1096	0.1254
CG	TRP	122	-0.1415	-0.0549	-0.0539	0.0196
CD1	TRP	122	-0.1638	0.0071	0.0114	0.1027
HD1	TRP	122	0.2062	0.1274	0.1332	0.1660
NE1	TRP	122	-0.3418	-0.4137	-0.3987	-0.3448
HE1	TRP	122	0.3412	0.3573	0.3789	0.4042
CE2	TRP	122	0.1380	0.0858	0.0788	0.1063
CZ2	TRP	122	-0.2601	-0.1119	-0.1184	-0.0625
HZ2	TRP	122	0.1572	0.1093	0.1215	0.1434
CH2	TRP	122	-0.1134	-0.1171	-0.1246	-0.0695
HH2	TRP	122	0.1417	0.0996	0.1045	0.1179
CZ3	TRP	122	-0.1972	-0.1238	-0.1311	-0.0952
HZ3	TRP	122	0.1447	0.0990	0.0911	0.1096
CE3	TRP	122	-0.2387	-0.1082	-0.1087	-0.0617
HE3	TRP	122	0.1700	0.0971	0.1010	0.1155
CD2	TRP	122	0.1243	-0.0348	-0.0401	-0.0207
C	TRP	122	0.5973	0.2690	0.2697	0.2659
O	TRP	122	-0.5679	-0.3756	-0.4292	-0.4119
N	GLY	123	-0.4157	-0.4420	-0.4216	-0.4198
H	GLY	123	0.2719	0.3401	0.3675	0.3688
CA	GLY	123	-0.0252	-0.0280	-0.0318	-0.0332
HA2	GLY	123	0.0698	0.1278	0.1310	0.1263
HA3	GLY	123	0.0698	0.1262	0.1325	0.1303
C	GLY	123	0.5973	0.2707	0.2743	0.2708
O	GLY	123	-0.5679	-0.3671	-0.3707	-0.3816
N	TRP	124	-0.4157	-0.4361	-0.4307	-0.4265
H	TRP	124	0.2719	0.3464	0.3620	0.3614
CA	TRP	124	-0.0275	0.0309	0.0362	0.0317
HA	TRP	124	0.1123	0.1275	0.1359	0.1328
CB	TRP	124	-0.0050	-0.1430	-0.1342	-0.1411
HB2	TRP	124	0.0339	0.1044	0.1171	0.1065
HB3	TRP	124	0.0339	0.1011	0.1349	0.1211
CG	TRP	124	-0.1415	-0.0436	0.0446	-0.0327
CD1	TRP	124	-0.1638	0.0131	0.1176	0.0234
HD1	TRP	124	0.2062	0.1326	0.1805	0.1445
NE1	TRP	124	-0.3418	-0.4114	-0.3353	-0.3970
HE1	TRP	124	0.3412	0.3531	0.4139	0.3791
CE2	TRP	124	0.1380	0.0934	0.1123	0.0909
CZ2	TRP	124	-0.2601	-0.1023	-0.0417	-0.1034
HZ2	TRP	124	0.1572	0.1104	0.1450	0.1180
CH2	TRP	124	-0.1134	-0.1062	-0.0341	-0.0982
HH2	TRP	124	0.1417	0.1046	0.1394	0.1138

CZ3	TRP	124	-0.1972	-0.1119	-0.0782	-0.1112
HZ3	TRP	124	0.1447	0.1018	0.1278	0.1062
CE3	TRP	124	-0.2387	-0.0993	-0.0364	-0.0926
HE3	TRP	124	0.1700	0.1064	0.1383	0.1181
CD2	TRP	124	0.1243	-0.0285	-0.0123	-0.0282
C	TRP	124	0.5973	0.2648	0.2617	0.2592
O	TRP	124	-0.5679	-0.3753	-0.3954	-0.4009
N	LEU	125	-0.4157	-0.4404	-0.4223	-0.4271
H	LEU	125	0.2719	0.3393	0.3584	0.3569
CA	LEU	125	-0.0518	0.0326	0.0309	0.0286
HA	LEU	125	0.0922	0.1244	0.1375	0.1327
CB	LEU	125	-0.1102	-0.1509	-0.1552	-0.1566
HB2	LEU	125	0.0457	0.0959	0.0946	0.0950
HB3	LEU	125	0.0457	0.0956	0.0954	0.0943
CG	LEU	125	0.3531	-0.0867	-0.0878	-0.0867
HG	LEU	125	-0.0361	0.0930	0.1015	0.1009
CD1	LEU	125	-0.4121	-0.2334	-0.2426	-0.2432
HD11	LEU	125	0.1000	0.0852	0.0868	0.0851
HD12	LEU	125	0.1000	0.0848	0.0856	0.0858
HD13	LEU	125	0.1000	0.0852	0.0850	0.0853
CD2	LEU	125	-0.4121	-0.2392	-0.2460	-0.2467
HD21	LEU	125	0.1000	0.0849	0.0848	0.0863
HD22	LEU	125	0.1000	0.0818	0.0854	0.0859
HD23	LEU	125	0.1000	0.0838	0.0870	0.0840
C	LEU	125	0.5973	0.2703	0.2699	0.2705
O	LEU	125	-0.5679	-0.3740	-0.3807	-0.3776
N	HIR	126	-0.4004	-0.4342	-0.4229	-0.4246
H	HIR	126	0.2872	0.3320	0.3542	0.3537
CA	HIR	126	0.0341	0.0393	0.0318	0.0327
HA	HIR	126	0.1034	0.1304	0.1299	0.1301
CB	HIR	126	-0.0309	-0.1346	-0.1362	-0.1392
HB2	HIR	126	0.0555	0.1157	0.1235	0.1218
HB3	HIR	126	0.0555	0.1174	0.1208	0.1211
CG	HIR	126	-0.0916	0.0950	0.0876	0.0829
ND1	HIR	126	-0.3663	-0.3719	-0.3616	-0.3664
HD1	HIR	126	0.3700	0.3688	0.4019	0.3968
CE1	HIR	126	0.0083	0.1802	0.1734	0.1740
HE1	HIR	126	0.2602	0.1605	0.1667	0.1617
NE2	HIR	126	-0.3504	-0.3704	-0.3694	-0.3675
CD2	HIR	126	-0.0209	0.0080	-0.0172	-0.0078
HD2	HIR	126	0.3046	0.1340	0.1326	0.1360
C	HIR	126	0.6126	0.2664	0.2562	0.2582
O	HIR	126	-0.5526	-0.3666	-0.3993	-0.3977
N	LEU	127	-0.4157	-0.4399	-0.4319	-0.4315
H	LEU	127	0.2719	0.3429	0.3554	0.3519

CA	LEU	127	-0.0518	-0.2847	-0.1048	-0.1061
HA	LEU	127	0.0922	0.1346	0.1034	0.1025
CB	LEU	127	-0.1102	-0.1102	-0.1102	-0.1102
HB2	LEU	127	0.0457	0.0457	0.0457	0.0457
HB3	LEU	127	0.0457	0.0457	0.0457	0.0457
CG	LEU	127	0.3531	0.3531	0.3531	0.3531
HG	LEU	127	-0.0361	-0.0361	-0.0361	-0.0361
CD1	LEU	127	-0.4121	-0.4121	-0.4121	-0.4121
HD11	LEU	127	0.1000	0.1000	0.1000	0.1000
HD12	LEU	127	0.1000	0.1000	0.1000	0.1000
HD13	LEU	127	0.1000	0.1000	0.1000	0.1000
CD2	LEU	127	-0.4121	-0.4121	-0.4121	-0.4121
HD21	LEU	127	0.1000	0.1000	0.1000	0.1000
HD22	LEU	127	0.1000	0.1000	0.1000	0.1000
HD23	LEU	127	0.1000	0.1000	0.1000	0.1000
C	LEU	127	0.5973	0.5973	0.5973	0.5973
O	LEU	127	-0.5679	-0.5679	-0.5679	-0.5679
Re	REQ	130	0.2760	0.7541	0.6880	0.6958
N1	REQ	130	-0.5515	-0.3808	-0.3680	-0.3630
CP0	REQ	130	-0.1051	0.0171	0.0436	0.0481
HP0	REQ	130	0.2729	0.1176	0.1188	0.1217
CP1	REQ	130	0.2258	-0.0900	-0.1171	-0.1120
HP1	REQ	130	0.0738	0.1104	0.1040	0.1058
CP2	REQ	130	-0.2327	0.0066	-0.0080	0.0018
CP2'	REQ	130	-0.4416	-0.2295	-0.2273	-0.2288
HP21	REQ	130	0.1786	0.0949	0.1016	0.0961
HP22	REQ	130	0.1786	0.0905	0.0970	0.0971
HP32	REQ	130	0.1786	0.0925	0.1017	0.0974
CP3	REQ	130	0.2336	0.0047	-0.0249	-0.0170
CP4	REQ	130	-0.2698	-0.0744	-0.1127	-0.1080
HP4	REQ	130	0.1770	0.1174	0.1126	0.1088
CP5	REQ	130	-0.2698	-0.0668	-0.1033	-0.1078
HP5	REQ	130	0.1770	0.1199	0.1123	0.1103
CP6	REQ	130	0.2336	-0.0048	-0.0255	-0.0253
CP7	REQ	130	-0.2327	0.0330	-0.0220	-0.0130
CP7'	REQ	130	-0.4416	-0.2131	-0.2279	-0.2270
HP71	REQ	130	0.1786	0.1027	0.0984	0.0972
HP72	REQ	130	0.1786	0.1060	0.0985	0.1022
HP73	REQ	130	0.1786	0.1084	0.1018	0.0992
CP8	REQ	130	0.2258	-0.0927	-0.1359	-0.1279
HP8	REQ	130	0.0738	0.1213	0.1146	0.1086
CP9	REQ	130	-0.1051	0.0778	0.0247	0.0354
HP9	REQ	130	0.2729	0.1349	0.1276	0.1282
N2	REQ	130	-0.5515	-0.3525	-0.3758	-0.3715
CP10	REQ	130	0.2109	0.1087	0.0841	0.0841

CP11	REQ	130	0.2109	0.0985	0.0824	0.0920
C1	REQ	130	0.2286	0.0733	0.0472	0.0397
O1	REQ	130	-0.1662	-0.1842	-0.2472	-0.2575
C3	REQ	130	0.2286	0.0829	0.0492	0.0483
O3	REQ	130	-0.1662	-0.1659	-0.2505	-0.2482
C2	REQ	130	0.2286	0.1156	0.0474	0.0558
O2	REQ	130	-0.1662	-0.1531	-0.2603	-0.2452

References

1. Shih, C.; Museth, A. K.; Abrahamsson, M.; Blanco-Rodriguez, A. M.; Di Bilio, A. J.; Sudhamsu, J.; Crane, B. R.; Ronayne, K. L.; Towrie, M.; Vlček, A., Jr.; Richards, J. H.; Winkler, J. R.; Gray, H. B., Tryptophan-Accelerated Electron Flow Through Proteins. *Science* **2008,** *320*, 1760-1762.

2. Lin, B.; Pettitt, B. M., On the universality of proximal radial distribution functions of proteins. *J. Chem. Phys.* **2011,** *134*, 106101

3. Polák, J.; Ondo, D.; Heyda, J., Thermodynamics of N-Isopropylacrylamide in Water: Insight from Experiments, Simulations, and Kirkwood−Buff Analysis Teamwork. *J. Phys. Chem. B* **2020,** *124*, 2495−2504.

4. Záliš, S.; Heyda, J.; Šebesta, F.; Winkler, J. R.; Gray, H. B.; Vlček, A., Photoinduced hole hopping through tryptophans in proteins. *Proc. Natl. Acad. Sci. U.S.A.* **2021,** *118*, 5775−5785.

5. Case, D. A.; Berryman, J. T.; Betz, R. M.; Cerutti, D. S.; III, T. E. C.; Darden, T. A.; Duke, R. E.; Giese, T. J.; Gohlke, H.; Goetz, A. W.; Homeyer, N.; Izadi, S.; Janowski, P.; Kaus, J.; Kovalenko, A.; Lee, T. S.; LeGrand, S.; Li, P.; Luchko, T.; Luo, R.; Madej, B.; Merz, K. M.; Monard, G.; Needham, P.; Nguyen, H.; Nguyen, H. T.; Omelyan, I.; Onufriev, A.; Roe, D. R.; Roitberg, A.; Salomon-Ferrer, R.; Simmerling, C. L.; Smith, W.; Swails, J.; Walker, R. C.; Wang, J.; Wolf, R. M.; Wu, X.; York, D. M.; Kollman, P. A. *AMBER 2014*, University of California: San Francisco, 2015.

6. Maier, J. A.; Martinez, C.; Kasavajhala, K.; Wickstrom, L.; Hauser, K. E.; Simmerling, C., ff14SB: Improving the Accuracy of Protein Side Chain and Backbone Parameters from ff99SB. *J. Chem. Theory Comput.* **2015,** *11*, 3696–3713.

7. Ponder, J. W.; Case, D. A., Force Fields for Protein Simulations. *Adv. Prot. Chem. 66,* **2003,** *66*, 27-85.

8. Zhang, Y.; Biggs, J. D.; Govind, N.; Mukamel, S., Monitoring Long-Range Electron Transfer Pathways in Proteins by Stimulated Attosecond Broadband X-ray Raman Spectroscopy. *J. Phys. Chem. Lett.* **2014,** *5*, 3656−3661.

9. Berendsen, H. J. C.; Grigera, J. R.; Straatsma, T. P., The Missing Term in Effective Pair Potentials. *J. Phys. Chem.* **1987,** *91*, 6269-6271.

10. Berendsen, H. J. C.; Postma, J. P. M.; van Gunsteren, W. F.; DiNola, A.; Haak, J. R., Molecular dynamics with coupling to an external bath. *J. Chem. Phys. 81,* **1984,** *81*, 3684-3690.

11. Heyda, J.; Pokorna, J.; Vrbka, L.; Vacha, R.; Jagoda-Cwiklik, B.; Konvalinka, J.; Jungwirth, P.; Vondrasek, J., Ion Specific Effects of Sodium and Potassium on the Catalytic Activity of HIV-1 Protease. *Phys. Chem. Chem. Phys.* **2009,** *11*, 7599−7604.

12. Ufimtsev, I. S.; Martínez, T. J., Quantum Chemistry on Graphical Processing Units. 3. Analytical Energy Gradients and First Principles Molecular Dynamics. *J. Chem. Theor. Comp.* **2009,** *5*, 2619-2628.
13. Titov, A. V.; Ufimtsev, I. S.; Luehr, N.; Martínez, T. J., Generating Efficient Quantum Chemistry Codes for Novel Architectures. *J. Chem. Theor. Comp.* **2013,** *9*, 213-221.
14. Hay, P. J.; Wadt, W. R., Ab initio effective core potentials for molecular calculations – potentials for K to Au including the outermost core orbitals. *J. Chem. Phys.,* **1985,** *82*, 299-310.
15. Hehre, W. J.; Ditchfield, R.; Pople, J. A., Self—Consistent Molecular Orbital Methods. XII. Further Extensions of Gaussian—Type Basis Sets for Use in Molecular Orbital Studies of Organic Molecules. *J. Chem. Phys.* **1972,** *56*, 2257-2261.
16. Adamo, C.; Barone, V., Toward reliable density functional methods without adjustable parameters: The PBE0 model. *J. Chem. Phys.* **1999,** *110*, 6158-6170.
17. Adamo, C.; Scuseria, G. E.; Barone, V., Accurate excitation energies from time-dependent density functionl theory: Assessing the PBE0 model. *J. Chem. Phys.* **1999,** *111*, 2889-2899.
18. Grimme, S.; Antony, J.; Ehrlich, S.; Krieg, H., A consistent and accurate ab initio parametrization of density functional dispersion correction (DFT-D) for the 94 elements H-Pu. *J. Chem. Phys.* **2010,** *132*, 154104.
19. Yanai, T.; Tew, D. P.; Handy, N. C., A new hybrid exchange-correlation functional using the Coulomb-attenuating method (CAM-B3LYP). *Chem. Phys. Lett.* **2004,** *393*, 51-57.
20. Ryckaert, J. P.; Ciccotti, G.; Berendsen, H. J. C., Numerical-Integration of Cartesian Equations of Motion of a System with Constraints - Molecular-Dynamics of N-Alkanes. *J. Comput. Phys.* **1977,** *23*, 327−341.
21. Mao, Y.; Montoya-Castillo, A.; Markland, T. E., Excited state diabatization on the cheap using DFT: Photoinduced electron and hole transfer. *J. Chem. Phys.* **2020,** *153*, 244111.
22. Voityuk, A. A.; Rösch, N., Fragment charge difference method for estimating donor–acceptor electronic coupling: Application to DNA π-stacks. *J. Chem. Phys.* **2002,** *117*, 5607-5516.
